# A lightweight neural attention-based model for service chatbots

**DOI:** 10.1038/s41598-025-14215-5

**Published:** 2025-08-13

**Authors:** Sinarwati Mohamad Suhaili, Mohamad Nazim Jambli

**Affiliations:** 1 Faculty of Cognitive Sciences and Human Development, Universiti Malaysia Sarawak, Kota Samarahan, 94300 Sarawak Malaysia; 2 Faculty of Computer Science and Information Technology, Universiti Malaysia Sarawak, Kota Samarahan, 94300 Sarawak Malaysia

**Keywords:** Attention mechanisms, Activation functions, Deep learning, Scalar functions, Score functions, Sequence-to-sequence, Weight initializer, Engineering, Mathematics and computing

## Abstract

The growing demand for efficient service chatbots has led to the development of various deep learning techniques, such as generative neural attention-based mechanisms. However, existing attention processes often face challenges in generating contextually relevant responses. This study introduces a lightweight neural attention mechanism designed to enhance the scalability of service chatbots by integrating a scalar function into the existing attention score computation. While inspired by scaling practices in transformer models, the proposed scalar is tailored to seq2seq architectures to optimize the alignment sequences, resulting in improved context relevance and reduced resource requirements. To validate its effectiveness, the proposed model was evaluated on a real-world Customer Support Twitter dataset. Experimental results demonstrate a +0.82 BLEU-4 improvement and a 28% reduction in training time per epoch over the baseline. Moreover, the model achieves the target validation loss two epochs earlier, indicating faster convergence and improved training stability. Further experiments investigated activation functions and weight initializers integrated into the proposed model to identify optimal configurations that optimize the model’s performance. Comparative experimental results show that the proposed modifications significantly enhance response accuracy and contextual relevance. This lightweight attention mechanism addresses key limitations of existing attention mechanisms. Future work may extend this approach by combining it with transformer-based architectures to support broader sequence prediction tasks, including machine translation, recommender systems, and image captioning.

## Introduction

Recent improvements in artificial intelligence (AI) and natural language processing (NLP) have significantly influenced the development of service chatbots. These developments enable companies to communicate with their clients effectively and at a lower cost, meeting the growing need for comprehensive automated customer service solutions. One of the most promising approaches in chatbot development is the use of seq2seq generative neural attention-based mechanisms. These models selectively focus on relevant parts of the input data, enabling more accurate and contextually appropriate responses. Despite these advances, notable challenges and limitations remain that must be addressed to achieve optimal performance.

In particular, existing attention-based seq2seq models struggle with context alignment; some input segments may be overlooked or misweighted, resulting in suboptimal responses and increased computational cost. To address these challenges, this study proposed a structural modification to the attention score function by integrating a scalar function. The goal is to create a lightweight solution that reduces computational overhead while improving the model’s ability to align input and output sequences through more effective learned attention weights. Existing attention mechanisms have not been extensively researched concerning the effects of different alignment score functions on the quality of responses generated. Moreover, the importance of accurately modeling the relationship between input and output in the attention layer remains inadequately addressed. This gap can result in misaligned or structurally irrelevant outputs, which compromise chatbot performance.

This study aims to address these limitations by examining the role of scaling-based normalization within attention mechanisms of seq2seq and its impact on the chatbot response quality. Specifically, the proposed scalar factor is expected to (i) mitigate gradient instability during training, (ii) refine the focus of attention mechanisms on the most relevant tokens, and (iii) improve the alignment between input queries and generated responses. To complement this structural modification, the model is further refined by evaluating various activation functions and weight initialization to quantify their impact on model performance..

The following are the main contributions of the proposed approach:

- We propose a lightweight structural modification to the seq2seq attention mechanism by incorporating a scalar function into the alignment score computation to generate better responses in the chatbot model.
- We analyze the impact of diverse activation functions and weight initialization schemes on model performance, identifying configurations that lead to improved model stability and efficiency.
- We conduct comparative experiments using a publicly available dataset to validate the proposed model’s performance against a baseline model, demonstrating its potential to enhance a service chatbot application.

The rest of the paper is organized as follows: sections “Related work” and “Method” provide the related work and overview of the proposed approach, respectively. Section “Experimental study” details the experimental study, including the dataset, experimental setup, and performance evaluation metrics used. The results and implications of these findings are discussed in section “Results and discussion” with recommendations for further research directions. Finally, section “Conclusion” summarizes the main contributions and highlights the significance of this study.

## Related work

Chatbots, also known as conversational agents, are AI-powered software applications that can interact with humans through natural language. These AI-driven applications have gained prominence in recent years as an efficient and cost-effective way for businesses to interact with their customers. In particular, service chatbots have been developed to perform various tasks, such as answering customer queries, providing product recommendations, and assisting with troubleshooting issues. Early chatbot systems adopted rule-based and template-driven models, evolving into machine learning and deep learning approaches that are either retrieval or generative-based designs^1–5^.

Seq2seq model using encoder-decoder (E2D) architectures has significantly advanced chatbot capabilities by generating relevant and coherent responses. Traditional modular chatbots comprising natural language understanding (NLU) and natural language generation (NLG), connected by a pipeline^6,7^, remain popular in industry due to their interpretability and stability^8^. However, there is growing research interest in (E2E) approaches based on seq2seq models, which integrate learning into a single neural framework^9^, inspired by neural machine translation^10^. These models are applicable across NLP tasks, such as chatbots, machine translation, recommendation systems, text summarization, image captioning, and sentiment analysis. While effective, traditional seq2seq models encounter difficulties with fixed-length vectors (context vectors), specifically when dealing with lengthy source sentences, where compressing the entire input sequence into a single vector degrades response quality^11^.

To address this issue, attention mechanisms were introduced by Bahdanau et al.^12^ and Luong et al.^13^, allowing the model to dynamically focus on relevant parts of the source sentence during decoding. This innovation improved translation and was adopted the chatbots. Several studies have built on these foundations. For example, Xing et al.^14^ implemented attention mechanisms in the encoder-decoder architecture to enhance question-answer relevance. The author proposes a novel model named Hierarchical Recurrent Attention Network (HRAN) to improve context-based response generation in conversational agents. HRAN uses a hierarchical attention mechanism to model the variance in the meaning of words and utterances in a unified framework. Another study by Yang & Tang^15^ introduces a short attention to speed up the attention mechanism’s computation using matrix transformation and convolution operation. The proposed method is suitable for more extended input and output lengths, where critical information is sparser, resulting in better performance. It can also compress the original sequence into a shorter sequence for subsequent processing, which makes it practical for long sentences in dialogue tasks. Further development incorporated conversation history and external knowledge from search engines as presented by Wang et al.^16^. The author enhanced chatbot informativeness by extracting relevant web data via the Rapid Automatic Keywords Extraction (RAKE) method for context enrichment. These approaches improve alignment and response accuracy but often remain computationally expensive.

Further research explores how model configurations influence attention performance. Suhaili et al.^17^ conducted a comparative study showing attention-based bi-RNNs paired with GloVe or FastText yielded better BLEU scores. Thorat & Jadhav^18^ proposed a model of Variational Hierarchical Conversational RNN with attention mechanism (VHCRA) to address the problem of the bypassing phenomena in VED, which could make the learning of variational space. Indirectly, it can overcome data degeneration problems and produce high-quality responses for a given context. Other studies introduced knowledge-aware attention^19,20^, but challenges of inconsistent context-response alignment persist.

In parallel with these seq2seq advancements, transformer-based architectures have emerged as a dominant force in NLP, particularly for open-domain dialogues and large-scale language understanding. Models such as GPT-3, GPT-4^21^, BERT^22^, and other ChatGPT-like systems rely on self-attention mechanisms to capture dependencies without the need for recurrent connections. Although transformer approaches often achieve state-of-the-art results in generating fluent and coherent responses, they come with notable computational overhead, due to the quadratic time complexity of self-attention^23^. This overhead may limit their practicality in resource-constrained environments.

As a result, there is strong motivation to refine attention mechanisms in seq2seq models. This work advances seq2seq attention-based mechanisms by integrating a scalar function into the attention score computation. This lightweight modification aims to narrow the performance gap between traditional seq2seq methods and resource-intensive transformer models, offering a scalable solution for service-oriented chatbots that demand both efficiency and contextual accuracy. Our model further examines how different activation functions and weight initializations influence training stability and performance, providing insights into optimizing attention mechanisms in resource-constrained environments. Table 1 summarizes related models, their approaches, and limitations, providing a concise comparison that highlights the motivation for our proposed modification.

**Table 1:** Summary of attention-based models and their limitations.

Model/Study	Attention mechanism	Strengths	Limitations
^12^	Additive Attention	Improves long-range alignment; dynamic scoring	Computationally more expensive than multiplicative
^13^	Multiplicative Attention	Efficient dot-product scoring	May underperform on longer sequences
HRAN (Chen et al.)	Hierarchical Attention	Captures both word and utterance-level context	Higher computational complexity
^15^	Short Attention	Speeds up long-sequence processing; efficient compression	May lose detailed context in compression
^17^	Bi-RNN + Attention	High BLEU scores; embedding-aware RNNs	Requires tuning embeddings and RNNs carefully
^16^	Context + Search + Attention	Uses search-engine data to enrich responses	Relies on external search precision and coherence
^19^	Hierarchical Attention + KB Match	Leverages knowledge base with semantic scoring	Dependent on entity linking quality
^18^	Variational Hierarchical Attention	Addresses VAE degeneration; better response diversity	Complex training; variational instability possible
^20^	Attention + Knowledge Graph + KVL	Handles OOVs; supports user intent with entities	Requires structured KG and memory models
Transformer (Vaswani et al.)	Scaled Dot-Product Self-Attention	Powerful contextual encoding; state-of-the-art results	Quadratic complexity; resource-intensive
Proposed model	Scalar-Normalized Additive Attention	Lightweight; improves alignment and convergence	Evaluated primarily on seq2seq chatbot tasks

## Method

This section describes the methodology employed in the study, which focuses on a hierarchical learning approach that utilizes the seq2seq learning model with the proposed neural attention mechanism. First, the problem description for the research is explained, followed by the overview of the proposed model and objective function, which are complementary mechanisms optimized by the network training environment.

### Problem descriptions

In the context of service chatbot applications, the task is to process an input request in a sequence of tokens $X = x_1, x_2, x_3, \ldots , x_m$ and produce a relevant output response as in the sequence of $Y = y'_1, y'_2, y'_3, \ldots , y'_n$, where the lengths of *X* and *Y* may be different. The primary goal is to determine the response with the highest conditional probability given the input question, which can be mathematically represented as maximizing *P*(*Y*|*X*). This maximization process is intuitive for human customer service agents with expertise, resulting in responses considered more “relevant” and helpful for a given question. However, in the realm of service chatbot systems, this process must be automated by learning a function *F*, parameterized by $\theta$, that maximizes the probability of a given input: $Y' = \arg \max F(X, \theta )$. To develop an effective chatbot application, the following key questions need to be addressed concerning the stated objective: (i) What is the structure of the model representing P(Y|X) in the context of service chatbots, and how can the proposed attention mechanism of score function with scaling factor be integrated to enhance the relevance between input questions and output responses? (ii) How to determine the optimal learning parameters $\theta$ to generate relevant responses? (iii) In terms of prediction, how to find the best $Y^\prime$ given *X* (input query) and $\theta$.

### Overview of the proposed model

**Fig. 1:**
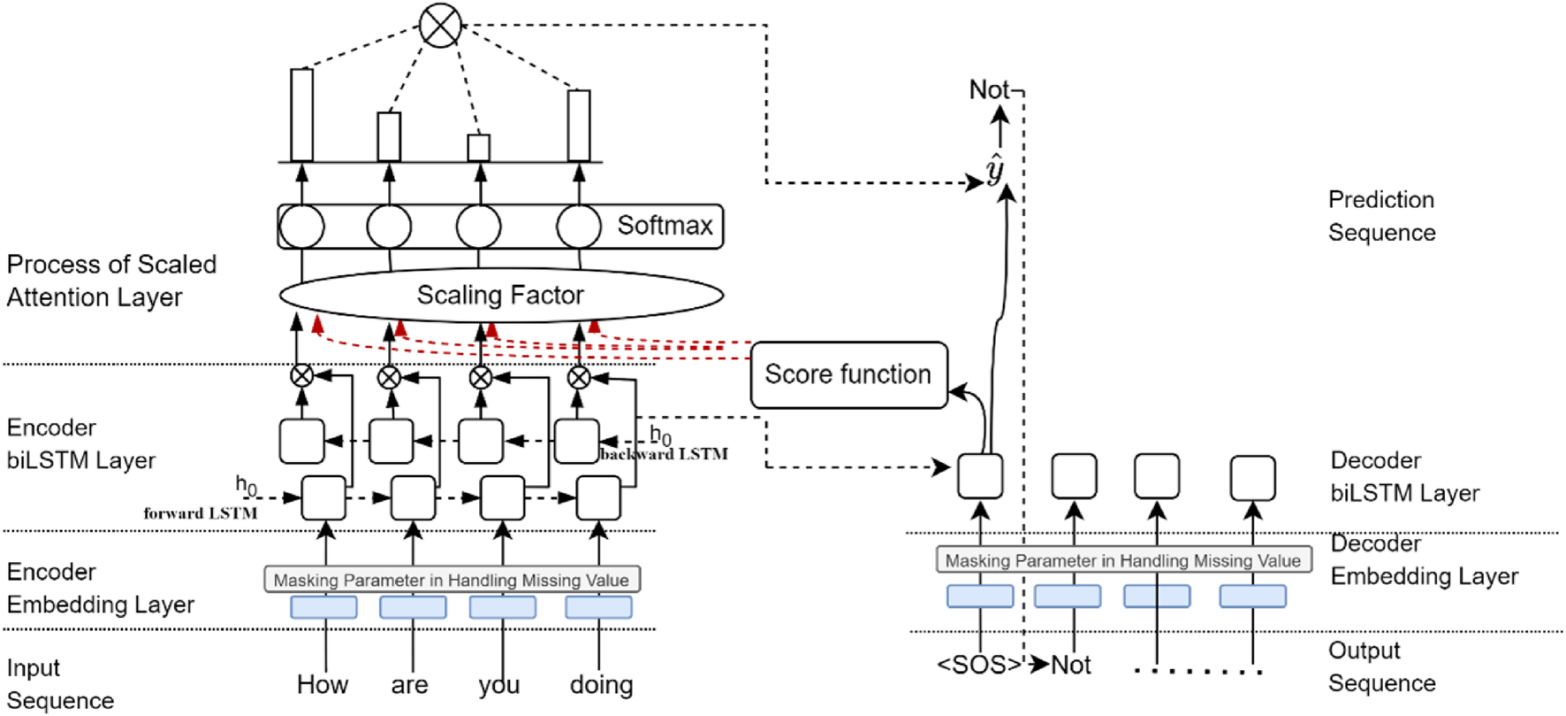
The proposed model’s architecture.

Figure 1 illustrates the overall architecture of the proposed model, which consists of four main components: an embedding layer, a biLSTM-based encoder within a seq2seq RNN structure for enhanced semantic and contextual learning of sequential input words, a decoder LSTM-based RNN for response prediction and the proposed neural attention score function for weighting the relevancy between queries and response relevance. Detailed descriptions of each component and their respective roles in the model are provided in the following subsections.

### Embedding layer

In this layer, we convert tokens to 300-d FastText vectors^24,25^ (alternatives include Word2Vec, GloVe). FastText, trained with a large corpus, is used to initialize the embedding layer. It is a highly efficient and flexible word representation technique that captures the semantic meaning of words through subword information. Using FastText embeddings, our model benefits from a rich representation of the input text, which helps improve the overall performance of the chatbot system. The embedding matrix is fixed during fine-tuning to focus learning on the proposed attention modification.

### Seq2Seq models

We employ a standard encoder–decoder (Seq2Seq) architecture with attention originally proposed by^10^. For each input sequence *X*, a bidirectional LSTM encoder produces hidden states $\textbf{h}_{1:m}$; the decoder LSTM then generates the target sequence *Y* token-by-token, conditioned on an attention-weighted context vector. Training maximises the conditional log-likelihood of *Y* given *X* with teacher forcing; inference uses either greedy decoding or beam search (section “Prediction of sequence”). A detailed overview of the Seq2Seq architecture is provided in Appendix A (Appendix A contains the loss derivation, the expanded LSTM equations, and the implementation details).

#### Attention layer

The previous section is based on the vanilla seq2seq model, where the encoder compresses all information into a single vector - a bottleneck problem that causes important information to be forgotten during the decoding step. To mitigate this problem, the model can be extended to include an added attention layer before the decoding step. Unlike the vanilla seq2seq model, the key concept behind attention is to use all the hidden states of the encoder network, since each hidden state contains information that can affect the decoder output at each timestep, rather than discarding the intermediate states of the encoder or using a single fixed context vector. Attention focuses differently on different words by assigning a score to each word. Then, using these softmaxed scores, the encoder’s hidden states are aggregated with a weighted sum of the encoder’s hidden states to obtain a context vector (*Ci*).

In the attention process, as detailed in Algorithm 1, the importance of each word in the input sequence is evaluated for every output cell. For each $y_{t}$ in the output *y*, its relevance is influenced by the context vector $c_{t}$ (source context for decoder step *t*) through an information filter applied to all hidden states $h = \left\{ h_{1},h_{2},h_{3},...,h_{m_{x}}\right\}$ of the encoder. Hence, the context vector $c_{t}$ can be calculated as in Eqs. (1–3).

1 $$\begin{aligned} c_{t} = \sum _{i=1}^{m_{x}}\alpha _{t_{i}}h_{i} \end{aligned}$$

where $\alpha _{t_i}$ is computed by

2 $$\begin{aligned} \alpha _{t_{i}} = \frac{exp(e_{ti})}{\sum _{j=i}^{m_{x}}exp(e_{tj})} \end{aligned}$$

where $e_{ti}=align(s_{t-1}, h_{i})$ is defined as the additive score function:

3 $$\begin{aligned} e_{ti}= V_{a}^{T}tanh(W_{a}s_{t-1}+U_{a}h_{i}) \end{aligned}$$

Here $\alpha _{ti}$ denotes the attention weights learned by the model, which determine the relevance of each input word, $W_{a}$, $U_{a}$ and $V_{a}$, a weighing parameter that must also be learned by the model through training. The alignment model (*align*) assesses the relationship between the input of position (*i*) and the output of the position (*t*) using a feedforward network that assigns higher values to related words and lower values to less associated ones. In addition, attention can capture the dependencies that are most important to the task, regardless of the item’s position.

**Algorithm 1:**
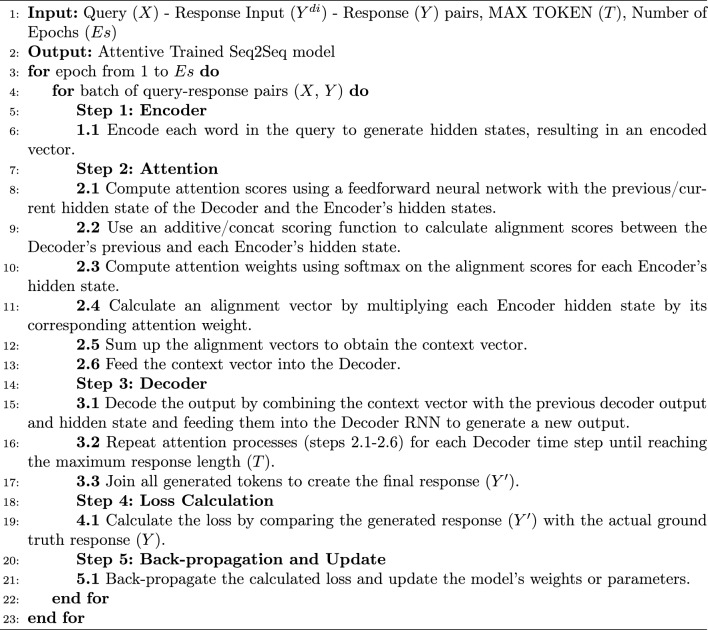
Attentive Seq2Seq model training for response generation.

#### Proposed method of attention mechanism modification

The augmentation of an attention layer, employing additive or concat scoring functions, was initially introduced by Bahdanau et al.^12^; he was the first author who pioneered the integration of the attention mechanism into the seq2seq model, as discussed previously. With the help of attention, the dependencies between source and target sequences are not constrained by the intervening distance. In this way, the model can learn how strong the relevance is between the source and the target, which can be computed by the attention-scoring function (ASF). However, the relevance between input and output in the attention layer is currently not being researched in detail, as some irrelevant structures may be present. Therefore, to address this limitation, we introduce a novel structural modification that incorporates a scaling factor into the computation of attention scores. The modification was made by adding a scaling factor to the existing equation of the score function: additive and multiplicative. The result of these score functions is divided by the scaling factor, which is the square root of the dimension of the source hidden state before applying the softmax function. This adjustment alters the way attention scores are calculated, resulting in a probability value that is normalized. The incorporation of a scaling factor into the attention score functions is inspired by the need to normalize the attention scores to stabilize the training process and improve model performance. The scaling factor $(\frac{1}{\sqrt{n}})$, is derived from the dimensionality of the input sequences, analogous to the normalization techniques used in other deep learning architectures, such as the scaling factor used in the dot-product attention mechanism introduced in the Transformer model^23^.

Normalizing attention scores has several benefits. For one, it keeps the gradients from becoming too large, which stabilizes training and enables faster convergence of the model. Moreover, this scaling factor ensures that the softmax function (which turns attention scores into probabilities) works within a range where it is not prone to overflow or underflow errors. Finally, using normalized scores allows the model to pay more attention to important parts of the input sequence thereby improving context understanding and overall performance. Relevance between source and target sequences refers to how well elements of a target sequence (the chatbot’s response) correspond to those in the source sequence (the user’s input). In terms of generative neural attention-based mechanisms, relevance is determined by attention scores used as weights for each input token in order to generate each output token. Higher attention scores on certain input tokens indicate their importance towards generating corresponding parts of an output sequence.

The correlation between the source and target sequences is crucial in determining the performance of a chatbot. By employing an attention mechanism to normalize this correlation, impressive results can be achieved in terms of chatbot performance. It can be seen that the chatbot generates responses that are more contextually accurate due to its enhanced understanding of the important aspects of the input. Furthermore, refining the attention scores leads to more logical and coherent output from the chatbot, thereby enhancing the overall user experience. Importantly, the proposed approach also facilitates faster learning, resulting in improved performance with fewer training iterations.

To further enhance the chatbot’s performance, we leverage various deep learning techniques, such as experimenting with different activation functions and weight initializers. Most of the study utilized the default Keras as the weight initializer, namely Xavier Glorot uniform. However, there are a few studies investigating the effects of other weight initializers, such as He_Normal and He_Uniform, on other activation functions in chatbot applications. The utilization of these techniques facilitates the optimization of the model’s parameters, leading to better results in terms of relevance, efficiency, and computational complexity. Moreover, a series of experiments were conducted to thoroughly investigate the relationship between various weight initializations, employing nonlinear activation functions other than the default Keras setting. This investigation further enhances our understanding of their impact on model performance.

Based on Algorithm 1 and Fig. 1, we address the attention layer part by focusing on the scoring function to highlight the work proposed in this study. Several common existing ASFs are implemented and investigated: additive/concatenate^12^, general/multiplicative^13^, and dot-product. This ASF was then improved by the proposed method mentioned earlier. In addition to multiplicative/general ASF, the modification was made by adding nonlinear activation into this function. The act refers to nonlinear activation functions such as hyperbolic tangent (tanh), rectified linear unit (ReLU), or scaled exponential linear unit (SELU), to name a few. In particular, the weights of the encoder output are randomly determined and learned during training by the backpropagation gradient process. This works with regard to global attention, as it produces more promising results than local ones^5,13^, wherein the entire source sequence is considered while generating each target token during the attention process. The steps are shown as follows:

1. ***Existing methods of attention scores function (ASF)***, *(*$e_{ti}=align(s_t, h_i)$*):* The attention score $e_{ti}$ in the attentive model can be computed using the attention methods as in Eqs. (4)–(6).
  *Dot product attention:*
    4 $$\begin{aligned} e_{ti} = s_t^\top \bar{h}_i \end{aligned}$$
    where $s_t$ is the decoder’s previous hidden state and $\bar{h}_i$ is the encoder’s hidden state.
  *Multiplicative (General) Attention:*
    5 $$\begin{aligned} e_{ti} = s_t^\top W_a \bar{h}_i \end{aligned}$$
    where $W_a$ is a learnable weight matrix.
  *Additive (Concatenate) Attention:*
    6 $$\begin{aligned} e_{ti} = v_a^\top \tanh (W_a s_t + U_a \bar{h}_i) \end{aligned}$$
    where $W_a$ and $U_a$ are weight matrices, and $v_a$ is a learnable vector.
2. **Proposed modifications of attention score function (ASF):** In addition to the standard multiplicative/general ASF, we introduce modifications by adding nonlinear activation functions. The activation function $\text {act}$ is applied to introduce nonlinearity into the attention score calculations, which can improve the model’s ability to capture complex relationships between the input and output sequences. The function $\text {act}$ refers to nonlinear activation functions used in the neural network layers, such as hyperbolic tangent (tanh), rectified linear unit (ReLU), or scaled exponential linear unit (SELU). The corresponding formulations for the modified multiplicative and additive attention mechanisms are presented in Equations (7) and (8).
  *Modified multiplicative attention:
    7 $$\begin{aligned} e_{ti} = \text {act}(s_t^T W_a \bar{h_i}) \end{aligned}$$
  *Modified additive attention
    8 $$\begin{aligned} e_{ti} = v_a^T \text {act}(W_a [s_t; \bar{h_i}]) \end{aligned}$$
  The existing, along with proposed modified attention scores (as indicated in *), incorporate a scaling factor into the attention score function to optimize the relevance between the source and target tokens. The modified attention score, denotes as $\bar{e_{ti}}$, is computed as shown in Eq. (9).
  9 $$\begin{aligned} \bar{e_{ti}} = f(e_{ti} *\frac{1}{\sqrt{n}}) \end{aligned}$$
  where the scaling factor is the square root of a dimension of the source hidden state, *n*. ***Interaction of scaling and activation.*** In the proposed variant, the modified attention scores include a normalisation factor $1/\sqrt{n}$, where $n$ is the hidden-state dimension. We first apply the nonlinear activation $\text {act}(\cdot )$ and then scale:
  $$e^{\text {raw}}_{ti} = \text {act}(\,\cdot \,), \qquad \bar{e}_{ti} = \frac{e^{\text {raw}}_{ti}}{\sqrt{n}}.$$
  The scaled logits $\bar{e}_{ti}$ are passed to the softmax as mentioned in Eq. (2), so that the variance of the pre-softmax distribution is bounded by $\sigma ^{2}/n$. Activating first reshapes the score distribution, while subsequent scaling keeps the logits in the linear region of $\operatorname {softmax}(\cdot )$, preventing gradient spikes. Empirically, this *activate-then-scale* strategy yields a BLEU improvement and smoother gradients, as described in section 5. The introduction of this scaling factor $\frac{1}{\sqrt{n}}$ normalizes the attention scores, which helps in stabilizing the training process, improving numerical stability, and enhancing the model’s ability to focus on the most relevant parts of the input sequence. For instance, if the dimensionality of the source hidden state is $n = 512$, the scaling factor would be $\frac{1}{\sqrt{512}} \approx 0.044$, ensuring that the attention scores remain within a manageable range.
3. ***Computation of attention scores and weights***: The attention score $e_{ti}$ is computed using the methods described above. The attention weights $\alpha _{ti}$ are then derived by applying the softmax function to the attention scores, as shown in Eq. (10).
  10 $$\begin{aligned} \alpha _{ti} = \frac{\exp (e_{ti})}{\sum _{j=1}^{m_x} \exp (e_{tj})} \end{aligned}$$
  The context vector, $c_t$, is obtained as a weighted sum of the encoder’s hidden states, as defined in Equation (11).
  11 $$\begin{aligned} c_t = \sum _{i=1}^{m_x} \alpha _{ti} \bar{h_i} \end{aligned}$$
4. ***Integration with the decoder***: The generated output sequence, $\hat{y}$, at each timestep of decoder LSTM is a candidate’s output, $\tilde{y}$, generated based on the previous decoder output, $\hat{y}_{j-1}$, and the previous hidden state, $s_{j-1}$, as formulated in Equation (12).
  12 $$\begin{aligned} \tilde{y} = \text {LSTM}^{\text {Dec}}(\hat{y}_{j-1}, s_{j-1}) \end{aligned}$$
  To incorporate the information from the source sequence, a weighted concatenation is performed that involves the context vector, $c_t$, and the candidate decoder output from the current timestep, as expressed in Equation (13).
  13 $$\begin{aligned} \text {concatenation} = [c_t, \tilde{y}] \end{aligned}$$
  The final attended output, $\hat{y}$, is generated as shown in Eq. (14).
  14 $$\begin{aligned} \hat{y} = W^c \cdot \text {concatenation} \end{aligned}$$
  where $W^c$ is a weight matrix used to determine the importance of the context vector and the candidate decoder output.

Having established the modified attention score $\bar{e_{ti}} = f(e_{ti} *\frac{1}{\sqrt{n}})$ and its scaling factor $\frac{1}{\sqrt{n}}$, we next analyse how this normalisation affects computational cost and training dynamics.

#### Computational complexity, gradient stability and convergence

The introduction of the scalar factor $1/\sqrt{n}$ prompts a natural concern regarding its computational overhead. Let *T* denote the length of the sequence of the source and *n* the dimensionality of the hidden state. For standard additive attention ^12^, the per-timestep floating-point operation count is given by Eq. (15).

15 $$\begin{aligned} F_{\text {add}}(T,n)=T\bigl (2n^{2}+3n\bigr )+\mathcal {O}(1), \end{aligned}$$

which accounts for two matrix–vector products and one $\tanh (\cdot )$ activation per timestep. Introducing the scalar factor $1/\sqrt{n}$^23^ adds only one fused multiply-add operation per token. The adjusted computational cost is therefore given by Eq. (15).

16 $$\begin{aligned} F_{\text {scaled}}(T,n)=F_{\text {add}}(T,n)+T =F_{\text {add}}(T,n)+\mathcal {O}(T), \end{aligned}$$

Given that all our experiments use hidden state dimensions $n\ge 128$, this additional $\mathcal {O}(T)$ remains insignificant relative to the dominant cost of $\mathcal {O}(T n^{2}).$

In addition to efficiency, scaling also offers numerical advantages. If attention logits $e_{ti}$ follow a normal distribution $\mathcal {N}(0, \sigma ^2)$, the scaled version $\tilde{e}_{ti} = e_{ti} / \sqrt{n}$ has reduced variance, $\operatorname {Var}(\tilde{e}_{ti}) = \sigma ^2 / n$. This reduction shifts the logits into the quasi-linear region of the $\operatorname {softmax}(\cdot )$ function, effectively bounding the Hessian ^8,23^. As a result, gradient magnitudes are better controlled, which supports more stable training and allows for a higher learning rate.

These properties result in fewer epochs to reach a given validation loss, which can be confirmed experimentally in section “Computational complexity, convergence and training-time analysis”.

### Prediction of sequence

In the context of response sequence prediction, the inference (decoding) phase plays a crucial role in utilizing the trained neural models to generate output sequences based on input sequences. This phase involves the sequential processing of input elements by the models, where the output of the previous timestep serves as the input for the current timestep. This autoregressive decoding continues until an end-of-sequence (eos) token is generated or a maximum length is reached. The trained models produce a probability distribution over the vocabulary at each timestep, from which the next token is selected. The quality of the generated output depends heavily on the decoding strategy used to traverse these distributions. In this study, we implement and evaluate three widely used inference methods8:

- **Greedy Search Decoding (GSD)**: selects the highest–probability token at every step.
- **Beam Search Decoding (BSD) (***k***)**: keep the best $k$ partial hypotheses and expand each one step-by-step until an end symbol is reached.
- **Beam Search with Length Penalty Decoding (BSLPD)**: extends beam search by incorporating a length-normalization term to avoid overly short or truncated output^26^ .

The beam search is approximately $k$ times slower than greedy decoding; therefore, we report the results for $k\!\in \!\{3,5\}$. Additional implementation details, including pseudocode and length-penalty formulation, are provided in Appendix B.

## Experimental study

This section presents a comprehensive experimental evaluation to assess the effectiveness of incorporating a scalar factor into the attention mechanism, as proposed in this work. The experimental study aims to address the following research questions:

- RQ1. Can the proposed model perform better than the baseline methods?
- RQ2. Is the proposed model sensitive to hyperparameters such as weight initializers and activation functions?
- RQ3. What are the insights and recommendations derived from the analysis of the proposed structural modification in chatbot attention mechanisms, and how can they inform future research and development in this area?

### Dataset

The experiment used the Customer Support on Twitter (CST) dataset from Kaggle, which was compiled in 2017. This dataset is a comprehensive and unique collection of tweets and responses that can be used for further natural language understanding (NLU) development and conversational models, as well as studying the modern customer service approaches and their effects. It includes 2,811,774 tweets/replies where 1,537,843(54.69%) were consumer-originated while 1,273,931(45.31%) were customer support agents originated. Out of the customer’s 1.5 million tweets; approximately 1.27 million received responses from support agents whereas around 0.23 million did not receive any replies. The CST dataset is an invaluable resource for researching customer service and NLU, containing authentic conversations between customers and support agents. These interactions feature natural responses from agents that accurately address problems and offer relevant solutions. Besides having a small message size which makes it practical for research purposes, especially recurrent neural networks.

**Fig. 2:**
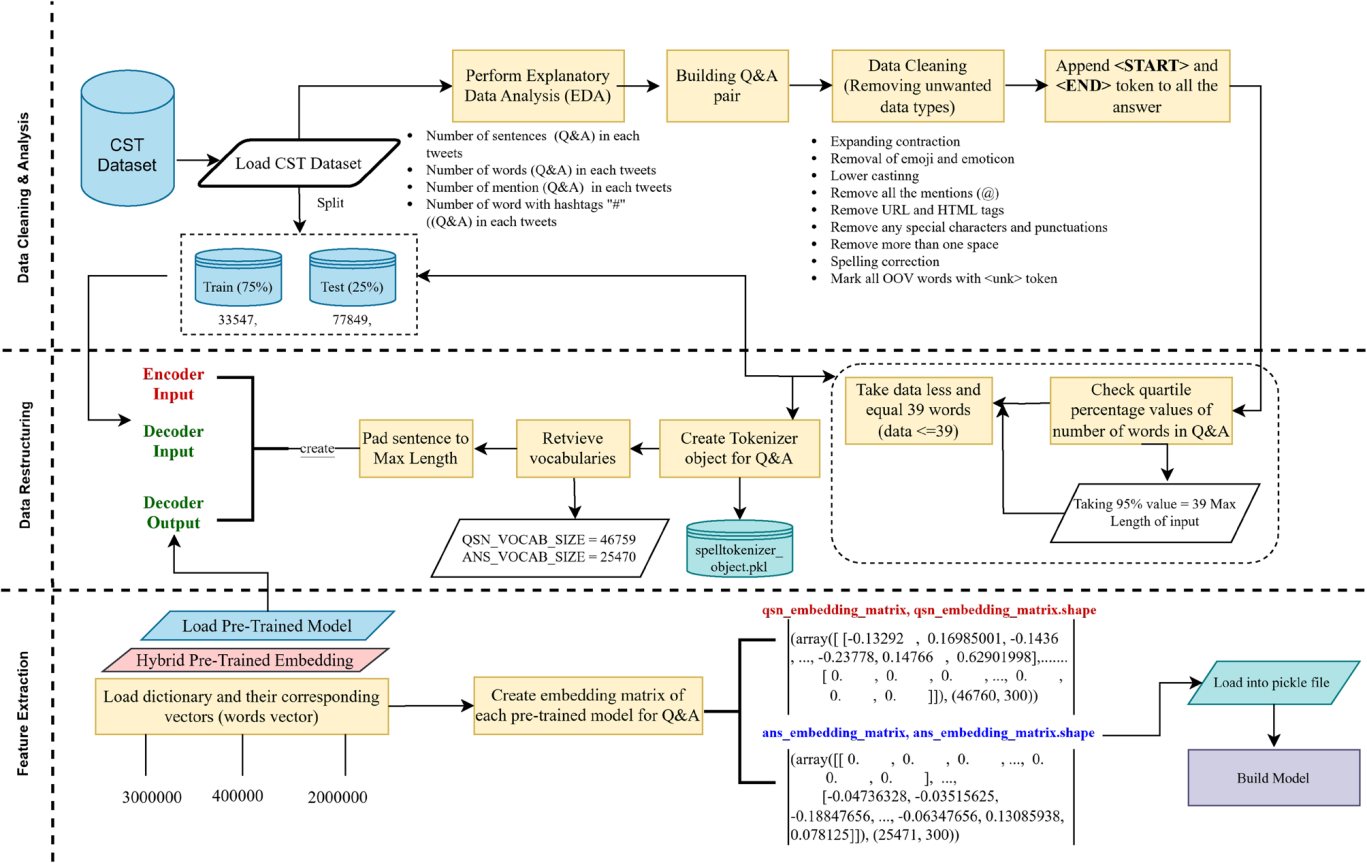
Overall process of data preparation for the CST dataset.

The dataset used as unstructured training data for the chatbot requires specific pre-processing steps to effectively prepare the data for subsequent chatbot processes as illustrated in Fig. 2. The pre-processing consists of two key steps: data cleaning and data restructuring. Various techniques are employed during the data cleaning step, including expanding contractions, removing emojis and emoticons, converting text to lowercase, eliminating mentions (words beginning with ‘@’) and URLs, removing words with digits, eliminating special characters and punctuation marks, correcting spellings, marking out-of-vocabulary words, and saving the cleaned text to disk. Exploratory data analysis is performed to gather insights into the dataset, including the number of words, common words, and word frequency.

Following data cleaning, the data restructuring process involves organizing the cleaned data into a suitable format for the encoder and decoder models. This includes appending ${<}$start${>}$ and ${<}$end${>}$ tokens to the decoder input (response), setting the maximum length of 39, creating records for encoder input, decoder input and decoder output, and selecting only the required columns.The dataset was randomly partitioned into training and validation subsets in a 75:25 ratio using a fixed seed (numpy.random.seed = 42) to ensure full reproducibility and eliminate temporal bias. Tokenization was performed to convert text into sequences of integers, with vocabularies constructed to map between words and indices. Token and emoji frequencies differed by no more than $\le 2\,\%$ across the splits, indicating no sampling bias was introduced. To manage sparsity, rare tokens (frequency < 3) were replaced with the special token ${<}$UNK${>}$. Furthermore, 300-dimensional pre-trained FastText embeddings were employed to encode sub-word information, enabling robust handling of informal, misspelled, or out-of-vocabulary words. Moreover, padding is applied to ensure uniform sequence lengths, and word embedding techniques are used to represent words as numerical vectors. By applying these pre-processing steps, the chatbot is equipped with clean and properly formatted data, which facilitates subsequent modeling and training processes for improved chatbot performance. The model is considered accurate when the predicted response aligns with the ground-truth answer. This study utilizes the Bilingual Evaluation Understudy (BLEU) score function to assess the performance of the models.

### Experimental setup

This study compares the proposed model with a baseline model, which consists of different attention-scoring functions to evaluate how well the models work. To ensure a fair comparison between the baseline and the proposed model, we initially compared the differences in the attention-scoring functions, including dot, multiplicative, and additive functions, to find the baseline model. The model showing the best performance in loss and BLEU score was chosen as a baseline for the proposed model. These models were implemented using a Python-dependent package within a deep neural network framework called TensorFlow^27^ and Keras. The training was conducted using a Jupyter Notebook hosted as an open-source platform called Google Colaboratory or Colab Pro+ with a high specification of memory, 89/50 GB RAM, and a GPU via subscription. A batch size of 64 was chosen, based on prior findings indicating its stability across training runs ^17^. The batch size is related to how many resources we have to accommodate the information for our model.

Moreover, we used a 300-dimensional FastText embedding in the input layer because its subword modeling consistently improved stability and BLEU performance in both LSTM and GRU encoders, as demonstrated in our prior work ^17^. The maximum length was determined by selecting 95-percentile values after performing EDA, i.e., 39 max_length. The architecture was selected by using a bidirectional LSTM in the encoder part and LSTM for the decoder part.

The hyperparameters and embedding configuration were guided by our previous empirical investigation ^17,28^, which demonstrated that RNN-based seq2seq models with 300 hidden units and 300-dimensional embeddings achieved stable and high BLEU scores. Extending these insights, we increased the hidden size to 480 units in this study to further enhance the model’s performance within our available memory constraints. This design choice aligns with prior research suggesting that increasing hidden dimensions improves model performance and generalization, especially for complex NLP tasks^29,30^.

Optimization was performed using the AdamW algorithm^31^ with a learning rate of 0.003 ^32^, consistent with configurations validated in our prior work^28^. In addition to improving a loss function, the optimizers are wrapped within Stochastic Weight Averaging (SWA) to achieve better generalization than conventional training^33^. In order to mitigate the issue of the ’exploding gradient’ problem, where gradients can rapidly increase to exponentially large values, resulting in unstable learning or even divergence, a gradient clipping value of 50.0 is implemented. This approach effectively prevents the cost function from overflowing with undefined values or overshooting cliffs. Furthermore, Xavier and Glorot uniform distributions^34^ are used as initializers for all weights and biases in Keras, as it is the default configuration.

Ablation studies, commonly conducted in deep learning research, are crucial for comprehending the impact of different components of our model. Our thorough analysis provides a clear understanding of how the activation function and weight initialization influence the stability and performance of service chatbots. Specifically, there is evidence to suggest that certain configurations result in more accurate answers which is crucial for maintaining high-quality responses in service chatbots. In addition, we included parameters such as BSD and BSLPD in the inference phase, with values of *k*=3, 5 and $\alpha$ = 0, 0.1, 0.5. By comparing ASF with other proposed models using a simulated experimental setup outlined in Table 2, we can demonstrate that ASF outperforms them.

**Table 2:** Experimental setup for the comparison of the standard variant of the ASF Seq2Seq model.

Parameter setting	Score function			
	M1-Dot	M2-General	M3-Additive	M4-Proposed
Max Length Input	39	39	39	39
Embedding size	300	300	300	300
Batch Size	64	64	64	64
Hidden Unit	480	480	480	480
Learning rate	0.003	0.003	0.003	0.003
Optimizer	AdamW	AdamW	AdamW	AdamW
Clipvalue	0.5	0.5	0.5	0.5
Word embedding	FastText	FastText	FastText	FastText
Encoder types	Bidirectional	Bidirectional	Bidirectional	Bidirectional
Activation of ASF			tanh	tanh, ReLU, Swish, Leaky_ReLU, Mish
Weight_Initializer	Glorot_Uniform	Glorot_Uniform	Glorot_Uniform	Glorot_Uniform, He_Uniform, He_Normal, Le_cun

### Evaluation metrics

This study primarily employs the BLEU metric ^20,35^, as suggested by Papelini (2010), to assess our model’s performance. According to Papelini ^36^, BLEU measures the co-occurrence of n-grams in the human reference translation and the model’s suggested responses. It calculates the n-gram precision for the entire dataset and includes a brevity penalty to penalize overly short translations. Following standard practice, we report BLEU-4, that is, $N=4$, which considers 1- to 4-gram matches. The more a candidate’s response overlaps with the reference across these *n*-gram orders, the higher the BLEU score, indicating closer similarity to human quality output.

Typically, the BLEU score is a metric for evaluating a machine translation system. In the context of a chatbot, the BLEU score compares the output text (hypothesis) generated by the chatbot with the response text (reference) generated by humans. It indicates how many *n*-grams of the output text are included in the reference. The more *n*-gram matches the candidate translation, the better it is. The BLEU score can take any value within the interval [0, 1] and is technically defined as Eq. (17).

17 $$\begin{aligned} BLEU = BP \times \left[ \prod _{n=1}^{N}precision_{n} \right] ^{1/N} \end{aligned}$$

Where *N* is the maximum *n*-gram number $n= [1,N]$ (N=4 in our evaluations). *BP* (brevity penalty) and $precision_{n}$ are defined in Eqs. (18) and (19), respectively.

18 $$\begin{aligned} BP = min (1,exp(1-\frac{ref_{lenght}}{out_{lenght}})) \end{aligned}$$

Where $ref_{lenght}$ is reference length, and $out_{lenght}$ is the chatbot output length.

19 $$\begin{aligned} precision_{n} = \frac{\sum _{n}^{EMPTY}min(m_{out}^{n},m_{ref}^{n})}{\sum _{n^{\prime }}^{EMPTY}m_{out}^{n^{\prime }}} \end{aligned}$$

Where $m_{out}^{n}$ is the number of *n*-grams matching the reference in the chatbot output, $m_{ref}^{n}$ denote as the number of *n*-grams in the reference, and $\sum _{n^{\prime }}^{EMPTY}m_{out}^{n^{\prime }}$ implies the total number of *n*-grams in the chatbot output.

The BLEU scores were calculated using the bleu score module from the translated package on the *nltk* platform, which was built in Python. Usually, the BLEU scores are used during the inference phase to evaluate the performance of the model. Depending on the interval in which the BLEU score values are located, the chatbot result can be interpreted as useless (if BLEU < 10), difficult to understand (10-19), understandable but with significant errors (20-29), average quality (30-40), high quality (40-50), very high quality (50-60), and better than a human (> 60). These values and interpretations were taken from the Google Cloud product description for AI and machine learning. However, by manipulating this reference score, in this study we categorized it as *“Good”*, *“Average”*, and *“Poor”* where the score less than or equal to 0.35 refers to the *“Poor”* category (x $\le$ 0.35), *“Average”* (x$\le$0.6), and greater than 0.6 (x$\ge$0.6) refers to the *“Good”* category.

## Results and discussion

In this section, we present the results of the proposed models in comparison to the baseline models for the CST dataset. The result of the baseline model is obtained by comparing the different ASF, including dot, multiplicative and additive, and will be used as a baseline for the proposed extension. Next, we report results for the Additive-based ASF and Multiplicative-based ASF with its proposed enhancements. Then, we make a comparative analysis between these two models and provide a qualitative analysis for selected models. The performance of the proposed model and the baseline models is reported in terms of loss and BLEU score. To enhance understanding of the discussions, a summary table (Table 3) is provided, listing the acronyms and terminology relevant to the studied models. This table serves as a guide, facilitating clear comparisons and qualitative analyses throughout this study.

**Table 3:** Key acronyms and terms defined for model analysis.

Model Abbreviation	Description
M1	Dot
M2	Multiplicative / General
M3	Additive /Concate
M4	Additive + Scaled Function + tanh
M4AR	Additive + Scaled Function + ReLU
M4ASW	Additive + Scaled Function + Swish
M4ALR	Additive + Scaled Function + Leaky_ReLU
M4AM	Additive + Scaled Function + Mish
M4AHT	Additive + Scaled Function + tanh—He_Uniform
M4AHR	Additive + Scaled Function + ReLU—He_Uniform
M4AHS	Additive + Scaled Function + Swish—He_Uniform
M4AHLR	Additive + Scaled Function + Leaky_ReLU—He_Uniform
M4AHM	Additive + Scaled Function + Mish—He_Uniform
M4ANR	Additive + Scaled Function + ReLU—He_Normal)
M4ALS	Additive + Scaled Function + SELU—Le_Cun
M5MA	Multiplicative + ACT(tanh)
M5MS	Multiplicative + Scaled Function
M5MAS	Multiplicative + ACT(tanh) + Scaled Function
M5MASR	Multiplicative+ ACT(ReLU) + Scaled Function
M5MASSW	Multiplicative + ACT(Swish)+ Scaled Function
M5MASLR	Multiplicative + ACT (Leaky_ReLU )+ Scaled Function
M5MASM	Multiplicative + ACT (Mish ) + Scaled Function
M5MAS_HU	Multiplicative + ACT (tanh) + Scaled Function
M5MASR_HU	Multiplicative + ACT(ReLU) + Scaled Function
M5MASS_HU	Multiplicative + ACT(Swish ) + Scaled Function
M5MASLR_HU	Multiplicative + ACT (Leaky_ReLU) + Scaled Function
M5MASM_HU	Multiplicative + ACT(Mish) + Scaled Function
M5MASR_HN	Multiplicative + ACT (ReLU) + Scaled Function
M5MASS_LC	Multiplicative + ACT (SELU) + Scaled Function
HU	He_Uniform
HN	He_Normal
LC	Le_Cun

### Baseline ASF results

Table 4 reports the result of a comparison between different ASF to determine the baseline. From this table, it can be seen that M2 and M3 perform comparably, with M2 performing better in validation loss during training, while M3-Additive performs well in the inference phase in both greedy and beam search, as evident from the resultant BLEU score. The difference in validation loss and greedy search BLEU between M2 and M3 is 0.21% and 0.09%, respectively. Therefore, these two models were selected as the base model for comparison with the proposed model for further investigation in this study.

**Table 4:** Result of ASF Seq2Seq models.

Model	Training phase		Inference phase	
	FASTTEXT			
	Loss	Val loss	BLEU	
			Greedy search	Beam search, k = 3
M1-Dot	1.072472	1.138756	0.436507	0.428405
M2-General	1.071377	1.136607	0.438087	0.431577
M3- Additive	1.073800	1.138623	0.438506	0.440251

### Additive-based ASF results

The enhancement results of the generative seq2seq models for an Additive-based ASF are presented in Table 5. The result shows that by including the scaling function, the enhancement model improved the performance of the model in terms of loss and BLEU score based on the greedy search technique.

**Table 5:** The result of the proposed models having additive attention with a scaling factor.

Model	Training phase		Inference phase	
	FastText			
	Loss	Val loss	BLEU	
			Greedy search	Beam search, k = 3
#M3-Additive	1.073800	1.138623	0.438506	0.440251
M4 - Additive + Scaled Function	1.072445	1.137282	0.438614	0.431225

In addition, the proposed model is extended by adding different activation functions to investigate the effects of activations on the proposed model, as shown in Table 6. The results show that the Leaky_ReLU activation with Glorot Uniform (GU) as the default weight initializer yields better loss performance. However, in the inference phase, the Greedy-based BLEU score was better with Swish activation, while the beam search size *k* = 3 with penalty $\alpha$ = 0 performs better with the Leaky_ReLU activation function.

**Table 6:** Second enhancement of additive ASF of seq2seq – Scaled Additive ASF + Activation + default initializer (Glorot Uniform).

Model	Loss	Val Loss	BLEU						
			Greedy Search	Beam search					
				k = 3	k = 5	$\alpha$ = 0, k = 3	$\alpha$ = 0.5, k = 3	$\alpha$ = 0, k = 5	$\alpha$ = 0.5, k = 5
#M4	1.072445	1.137282	0.438614	0.431225					
M4AR	1.069883	1.136072	0.436501	0.431688	0.433956	0.444409	0.441762	0.432170	0.438291
M4ASW	1.073854	1.137080	**0.438971**	0.432181	0.436614	0.433565	0.434902	0.436234	0.431222
M4ALR	1.071599	**1.135726**	0.437864	0.428708	0.435355	**0.446262**	0.430031	0.434155	0.433872
M4AM	1.070414	1.136258	0.438955	0.433454	0.436427	0.441877	0.442067	0.427182	0.434949

Significant values are in [bold].

The proposed model was then improved by different weighting initializations and activation functions, including He_Uniform, He_Normal, and Le_Cun, as indicated in Table 7. From this table, it can be seen that Mish activation with He_Uniform performs better in loss minimization during training, while SELU activation with Le_Cun as the weight initializer performs better in the inference phase in both greedy and beam search with size *k*=3 and penalty $\alpha$ = 0.

**Table 7:** Second enhancement of additive ASF of seq2seq – Scaled Additive ASF + activation + Additional WI.

Model	Loss	Val loss	BLEU						
			Greedy search	Beam search					
				k = 3	k = 5	$\alpha$ = 0, k = 3	$\alpha$= 0.5, k = 3	$\alpha$ = 0, k = 5	$\alpha$ = 0.5, k = 5
*M4AHT	1.071200	1.138650	0.436598	0.432235	0.441807	0.446180	0.439677	0.441469	0.436208
M4AHR	1.070105	1.135668	0.439154	0.451428	0.442181	0.437486	0.437808	0.433220	0.440940
M4AHS	1.070025	1.136831	0.439057	0.434026	0.434784	0.432526	0.439302	0.435828	0.439410
M4AHLR	1.070037	1.135566	0.437460	0.435924	0.436633	0.451548	0.430097	0.425145	0.435059
M4AHM	1.070243	**1.134345**	0.439197	0.441446	0.436521	0.438819	0.427420	0.432288	0.440766
M4ANR	1.074149	1.140075	0.436894	0.438861	0.431307	0.431307	0.441283	0.438861	0.433997
M4ALS	1.070615	1.136054	**0.440779**	0.438271	0.438186	**0.459270**	0.440892	0.433620	0.442607

Significant values are in [bold].

In essence, different activation functions and weight initialization methods lead to slight variations in the performance of the models. It appears that the M4AHM model (Mish activation, He_Uniform initialization) performs relatively well in terms of validation loss and consistently across different search methods. However, the M4ALS model (SELU activation, Le_Cun initialization) achieves the highest BLEU score in some configurations. This suggests that different combinations of activation functions and weighting initialization methods could lead to slightly improved results, but none appear to provide a significant advantage over others under all conditions. In other words, there is no clear ”best choice” for all scenarios, and the optimal combination might depend on the specific requirements and constraints of the task.

### Multiplicative-based ASF results

The result of multiplicative-based enrichment by individually including the activation function (act) and the scaling function, as well as a combination of both, is shown in Table 8. The results show that the combination of both activations with the scaling function gives promising results for both loss and BLEU scores based on greedy and beam searches with size *k* = 3. The M5MAS model, which incorporates both of these enhancements, consistently achieves the highest BLEU scores across all tested conditions. Thus, this model (M5MAS) was used as an indicator for the following improvement approaches.

**Table 8:** Enhancement of multiplicative/general ASF of seq2seq – Scaled Multiplicative ASF.

Model	Loss	Val loss	BLEU						
			Greedy search	Beam search					
				k = 3	k = 5	$\alpha$ = 0, k = 3	$\alpha$ = 0.5, k = 3	$\alpha$ = 0, k = 5	$\alpha$ = 0.5, k = 5
#M2	1.071038	1.137435	0.438132	0.430391					
M5MA	1.070897	1.136484	0.438926	0.444001	0.432404	0.438130	0.435834	0.436026	0.442150
M5MS	1.071097	1.137715	0.437876	0.436203	0.431859	0.435222	0.430836	0.440271	0.432507
M5MAS	1.071383	**1.136370**	**0.440390**	**0.444001**	0.432404	0.438130	0.435834	0.436026	0.442150

Significant values are in [bold].

The following improvements are made by extending multiplicative-based models with the integration of different activation functions (ReLU, Swish, Leaky_ReLU, Mish) and standard weight initialization (WI). The result reveals that the base model performs better than the other activation functions, indicating future use for the following improvements, as presented in Table 9. Moreover, the results of incorporating various initializers, including He_Uniform, He_Normal, and Le_Cun (other than Glorot Uniform), in extended multiplicative-based seq2seq models are shown in Table 10. The results show that ReLU activation with the He_Normal initializer performs better in loss minimization, while in the inference phase, the Mish activation with the He_Normal initializer shows better performance in terms of BLEU score based on greedy and beam search with a size of *k*=5 and a penalty ($\alpha$) of 0.5. One pattern to notice is that models using He_Uniform initialization generally achieve higher BLEU scores compared to those using He_Normal (HN) and Le_Cun (LC), suggesting promising use for future work.

**Table 9:** Enhancement of multiplicative/general ASF of seq2seq – Scaled Non-Linearity Multiplicative ASF + WI (Default initializer).

Model	Loss	Val loss	BLEU						
			Greedy search	Beam search					
				k = 3	k = 5	$\alpha$ = 0, k = 3	$\alpha$ = 0.5, k = 3	$\alpha$ = 0, k = 5	$\alpha$ = 0.5, k = 5
#M5MAS	1.071383	**1.136370**	**0.440390**	**0.444001**	0.432404	0.438130	0.435834	0.436026	0.442150
M5MASR	1.071169	1.136459	0.437862	0.435527	0.442034	0.436481	0.434426	0.435095	0.432537
M5MASSW	1.075163	1.138702	0.437833	0.439691	0.437954	0.435338	0.427973	0.430772	0.442916
M5MASLR	1.073412	1.137750	0.436906	0.433256	0.431691	0.430406	0.434816	0.426934	0.435159
M5MASM	1.072167	1.137465	0.435777	0.429783	0.443128	0.430794	0.437888	0.441483	0.430361

Significant values are in [bold].

**Table 10:** Enhancement of multiplicative/general ASF of seq2seq – Scaled Scaled Non-Linearity Multiplicative ASF + WI (others initializer instead of GU).

Model	Loss	Val loss	BLEU						
			Greedy Search	Beam search					
				k = 3	k = 5	$\alpha$ = 0, k = 3	$\alpha$ = 0.5, k = 3	$\alpha$ = 0, k = 5	$\alpha$ = 0.5, k = 5
M5MAS_HU	1.070049	**1.135154**	**0.438430**	**0.432948**	0.439174	0.440727	0.427681	0.427286	0.437871
M5MASR_HU	1.070795	1.136159	0.439681	0.432827	0.434364	0.435472	0.438578	0.439992	0.435305
M5MASS_HU	1.072608	1.137611	0.439744	0.437104	0.440938	0.433388	0.437800	0.437104	0.443095
M5MASLR_HU	1.071469	1.137303	0.437475	0.435840	0.436777	0.445197	0.431935	0.432465	0.441057
M5MASM_HU	1.070757	1.135852	**0.440445**	0.435013	0.440360	0.445040	0.433824	0.433408	**0.445236**
M5MASR_HN	1.069998	**1.134963**	0.439069	0.433964	0.438484	0.438871	0.429469	0.428625	0.439606
M5MASS_LC	1.071313	1.138230	0.437069	0.442683	0.440404	0.436702	0.429345	0.429440	0.436679

Significant values are in [bold].

### Computational complexity, convergence and training-time analysis

Having established the accuracy improvements, we next examine whether the scalar normalisation ${1}/{\sqrt{n}}$ also provides the anticipated efficiency gains. Table 11 presents the average wall-clock training time per epoch for the additive baseline (#M3) and its scaled variant (M4). The scalar version (M4) achieves a training speed-up of approximately **28 %**, reducing the average epoch time from 4,830 seconds to 3,480 seconds on the specified hardware.

**Table 11:** Average epoch time on the CST dataset.

Model	Time/epoch (s)	Relative $\Delta$
#M3 Additive	4830	–
M4 Additive + Scaled Function	3480	– 28 %

**Fig. 3:**
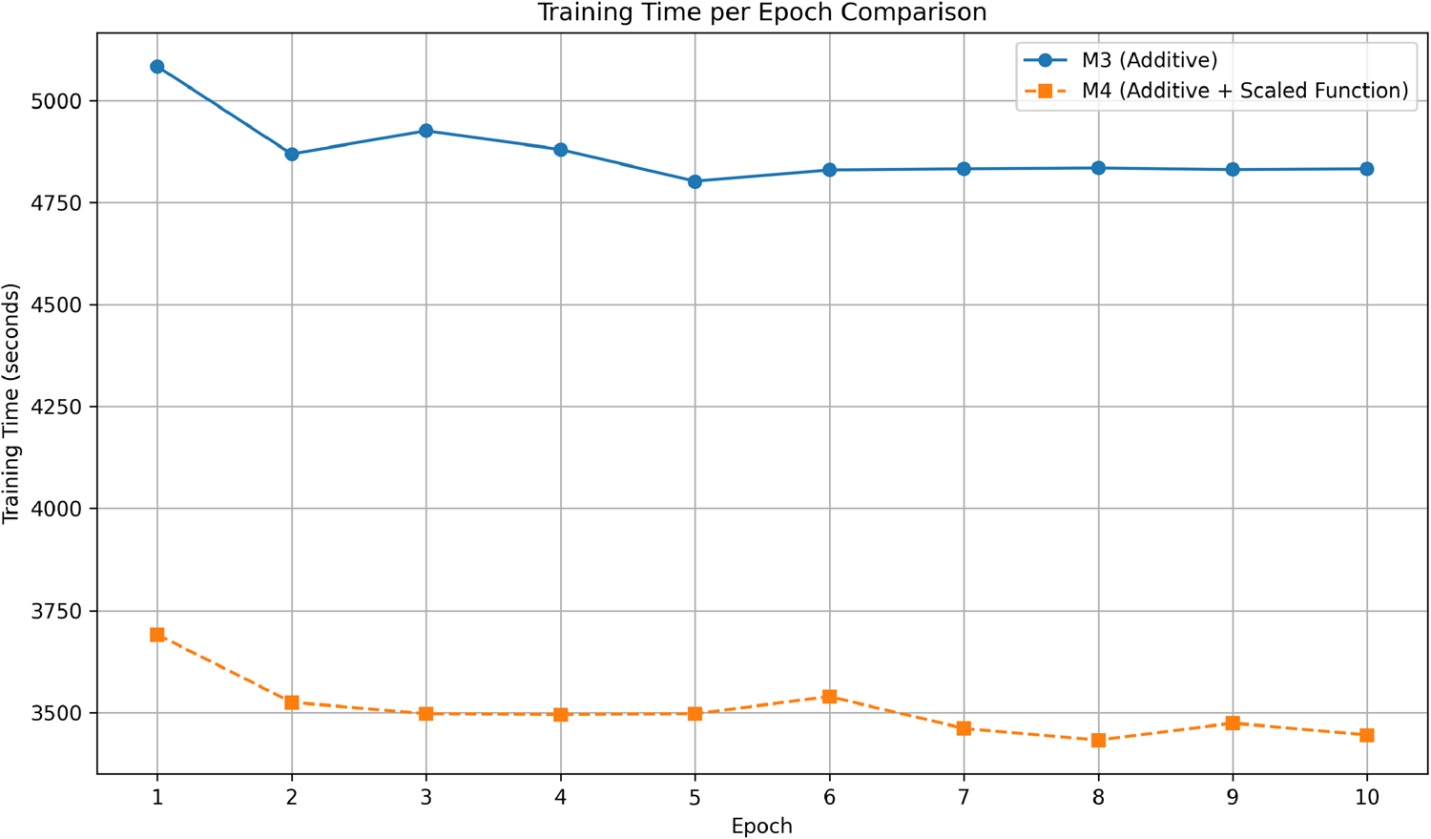
Training-time per epoch on the CST dataset for the additive model without (blue) and with (orange) the scalar factor.

To complement these findings, Fig. 3 compares the average wall-clock time per epoch for the additive baseline (#M3) and its scalar–normalised counterpart (M4). The inclusion of the scalar factor reduces the epoch duration from 5084 s to 3480 s on average (a reduction of approximately **28%**). This improvement confirms that the $1/\!\sqrt{n}$ normalisation effectively decreases computational complexity, particularly by reducing the overhead associated with matrix multiplication in attention score computations.

**Fig. 4:**
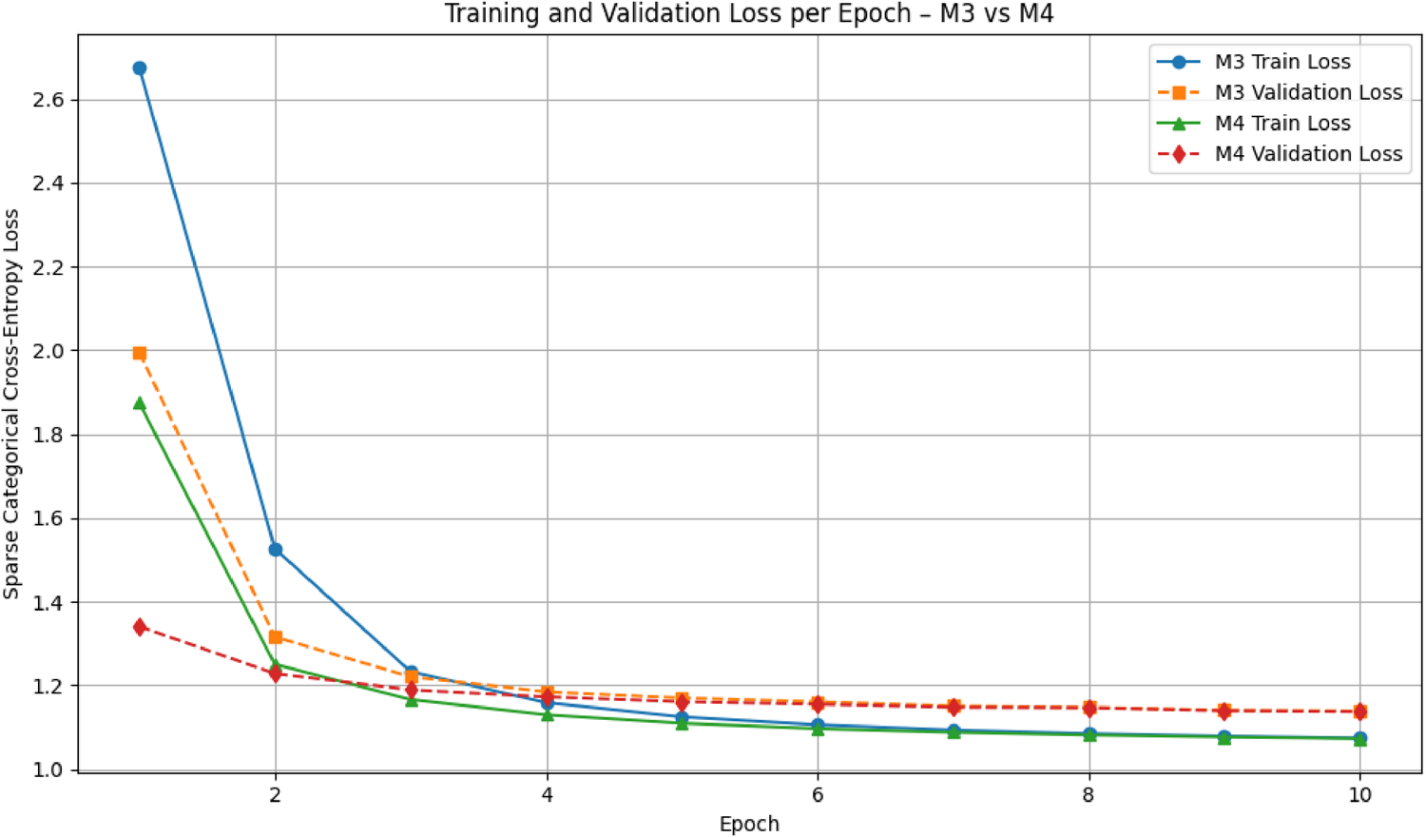
Training and validation loss per epoch for the baseline additive attention model (#M3) and the proposed scaled-attention model (M4).

Figure 4 shows the training and validation loss curves for the base model of additive attention (M3) and the proposed variant of scaled attention (M4), trained for 10 epochs using the sparse categorical cross-entropy losses. The number of training epochs was intentionally limited due to computational resource constraints.. In general, M4 shows clear advantages in both convergence speed and training stability. It starts with substantially lower initial losses (training $\approx$ 1.88, validation $\approx$ 1.34) compared to M3 (training $\approx$ 2.67, validation $\approx$ 1.99), indicating that the scalar normalization leads to more favorable initial parameter conditions. In epoch 3, M4 achieves a validation loss of less than 1.20, while M3 only reaches comparable values in epoch 5, which illustrates a more efficient progression through the optimization environment.

In addition to faster convergence, M4 shows a smoother and more stable learning curve compared to M3, with training and validation losses steadily decreasing over epochs. In contrast, M3 shows larger fluctuations, especially in early training, indicating less stable optimization dynamics. The consistent progression observed in M4 reflects more controlled gradient behavior and stronger generalization to unseen data, likely facilitated by the scalar-normalized attention mechanism. This consistent convergence supports the performance gains observed in BLEU-4 (see Table 12) and strengthens the proposed adjustment.

In a holdout batch comprising 1000 samples, we measured the end-to-end decoding latency and evaluated translation quality (see Table 12). The scaled model (M4) achieves an average decoding latency of **0.272 s** per sentence, representing approximately a 12% reduction in decoding time compared to the baseline model (#M3), while simultaneously improving the BLEU score from 0.4379 to 0.4417. Thus, the scalar normaliser yields lighter computation both at training and at inference time without compromising output quality.

**Table 12:** Inference speed and quality on 1000 CST validation utterances.

Model	BLEU $\uparrow$	Time/sample $\downarrow$ (s)
#M3 – Additive (no scaling)	0.4379	0.308
M4 – Additive + scaled function	**0.4417**	**0.272**

Significant values are in [bold].

**Fig. 5:**
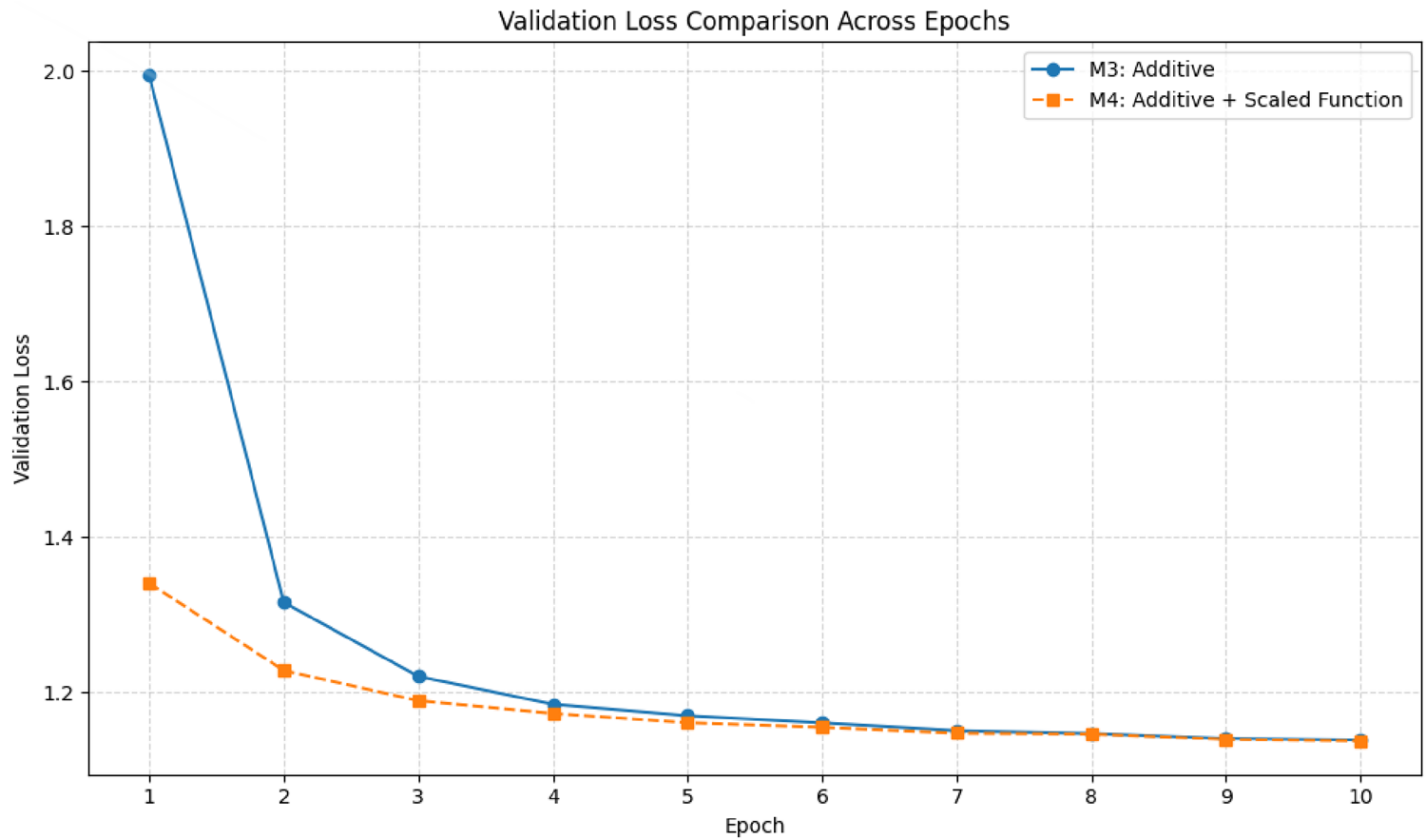
Validation loss per epoch for the baseline additive attention model (#M3) and the proposed scaled-attention model (M4).

Figure 5 compares validation loss between the baseline and scalar model (M4). M4 begins training at a significantly lower validation loss of 1.34 compared to the baseline’s 1.99, and reaches a loss of 1.14 two epochs earlier. Moreover, its loss trajectory is noticeably smoother, indicating reduced variance during training. This empirical observation supports the theoretical analysis provided in section “Seq2Seq models”, highlighting how the scalar factor maintains activation values within a numerically stable range, thus enabling faster and more consistent convergence.

### Comparative analysis between models

The performance comparison between the proposed model and the baseline model is presented as a comparative analysis. The performance of the model is evaluated by the validation loss in the training phase, and the BLEU score is used in the inference phase. Since this experiment is very computationally intensive, we only tested Glorot Uniform and He_Uniform weight initializations (WI) for similar activation functions. The comparative analysis of the models is explained and divided into categories. The first one is based on the additive-based model, the next one is based on the multiplicative-based model and, finally, all the models were compared with the baseline and the performance of the proposed method was evaluated by statistical significance tests as follows:

#### Comparative base model with enhanced additive ASF

**Fig. 6:**
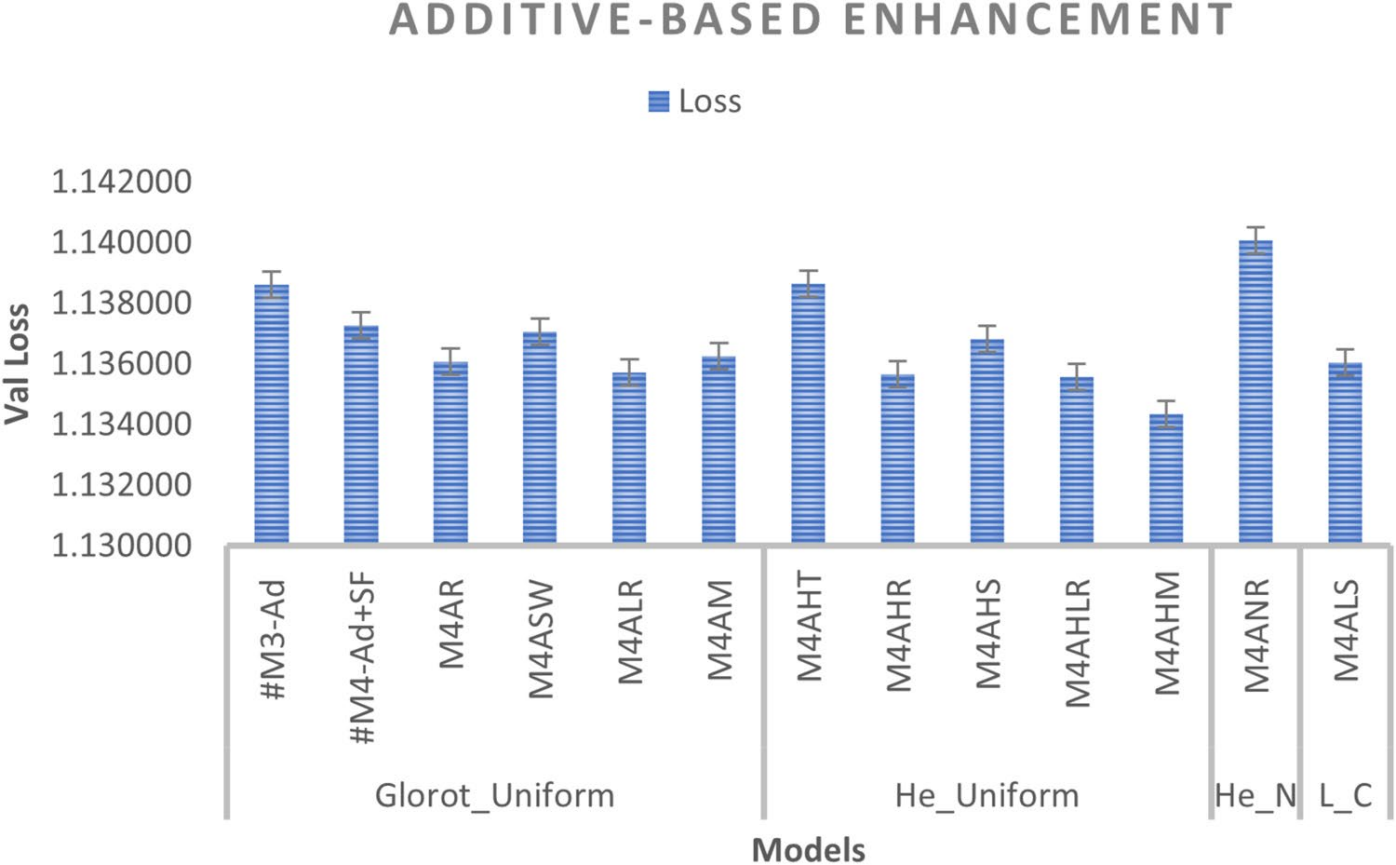
Additive-based enhancement (performance during training phase).

**Fig. 7:**
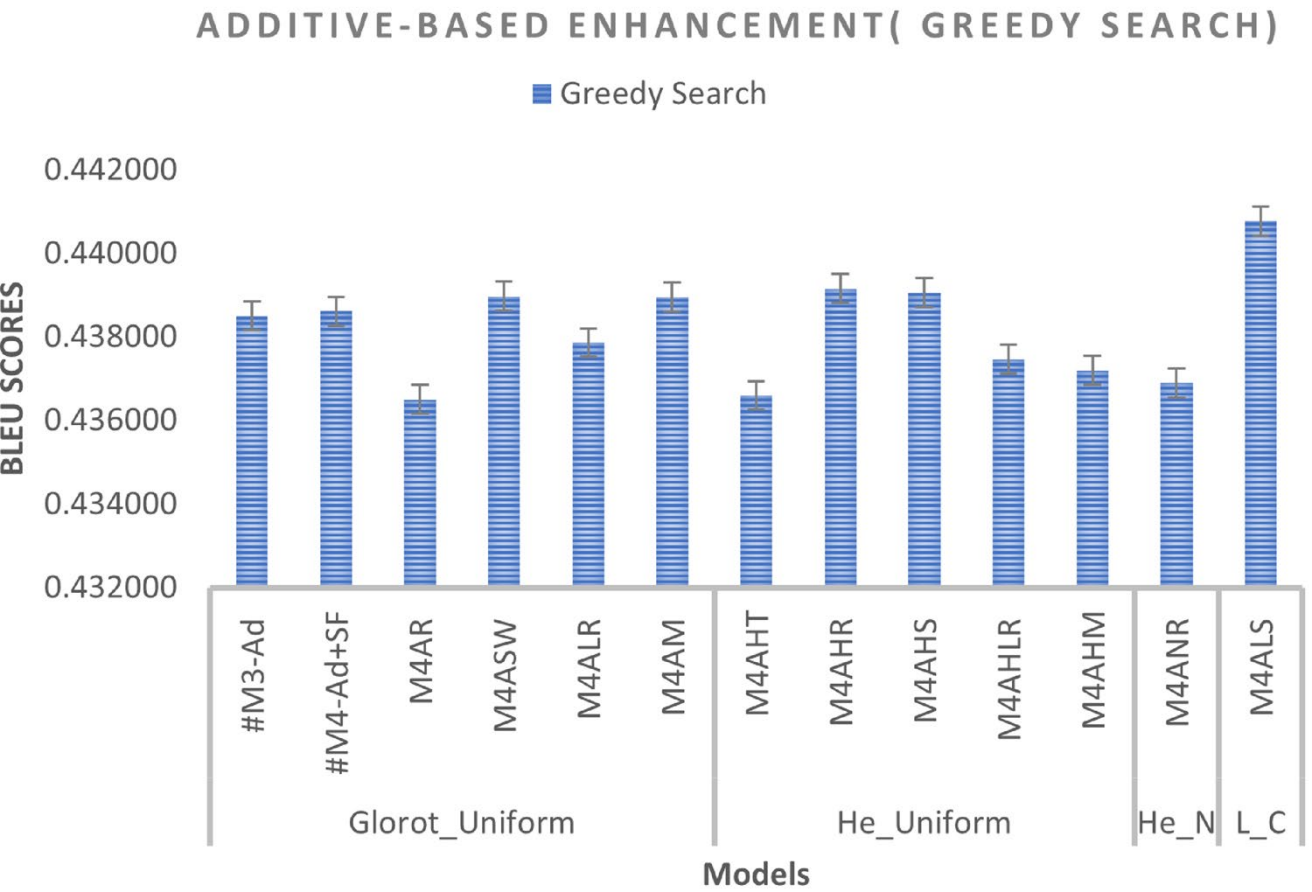
Additive-based enhancement (performance during inference phase – Greedy search prediction).

**Fig. 8:**
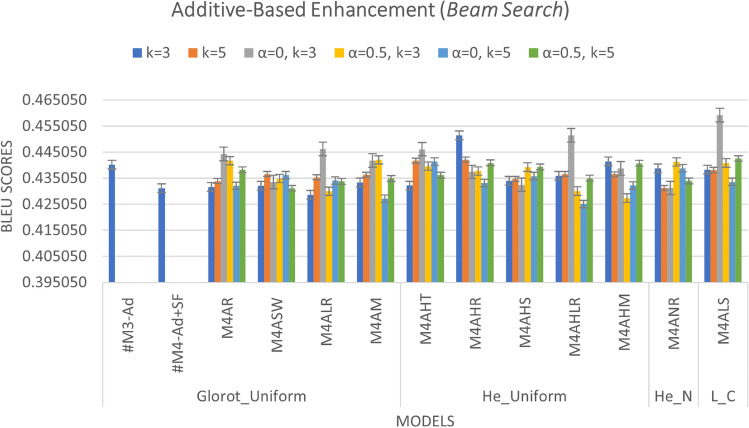
Additive-based enhancement (performance during inference phase – beam search prediction)).

We added a scalar factor to the additive-based models with different WI and activation functions and compared it with the base model. As can be seen in Figs. 7 and 8, the M4ALS model achieves the best result in BLEU score performance for both greedy and beam search with parameter size 3 and penalty 0 ($\alpha$ = 0, *k* = 3), whereas in training, the M4AHM model achieves the lower loss compared to #M3-Ad (base model), as shown in Fig. 6.

*Effect of WI*: We compare the standard weight (Glorot_Uniform) initialized in Keras with other WI, including He_Uniform, He_Normal and Le_Cun. As can be observed, most models obtain better results with the initializers He_Uniform and Le_Cun, with 42 per cent of the total experiments for each of the different models showing better results, suggesting that this weight initializer provides promising results for future work.

*Effect of activation functions*: We compare the default activation (tanh) used in Keras with other activation functions such as ReLU, Swish, Leaky_ReLU, Mish, and SELU, as shown in Fig. 8. The result indicates that the SELU activation functions with the Le_Cun initializer as the most frequent model give the best results for greedy and beam search prediction. In addition, the ReLU activation function with H_Uniform, instead of the Glorot Uniform initializer, can also be chosen as it gives the second-best result in creating a better model.

*Effect of beam search parameters*: In the inference phase, we tested different beam search parameters in terms of size (*k*) and penalty ($\alpha$). As illustrated in Fig. 8, the result is difficult to interpret because it varies according to the different predefined parameters. However, it can be seen that the beam size parameters *k* = 5 and penalty $\alpha$ = 0 were the most common and gave promising results among the models. Meanwhile, this parameter achieves the best results when the Le_Cun initializer is used with the SELU activation function, indicating that it is promising for future work.

#### Comparative base model with enhanced multiplicative ASF

**Fig. 9:**
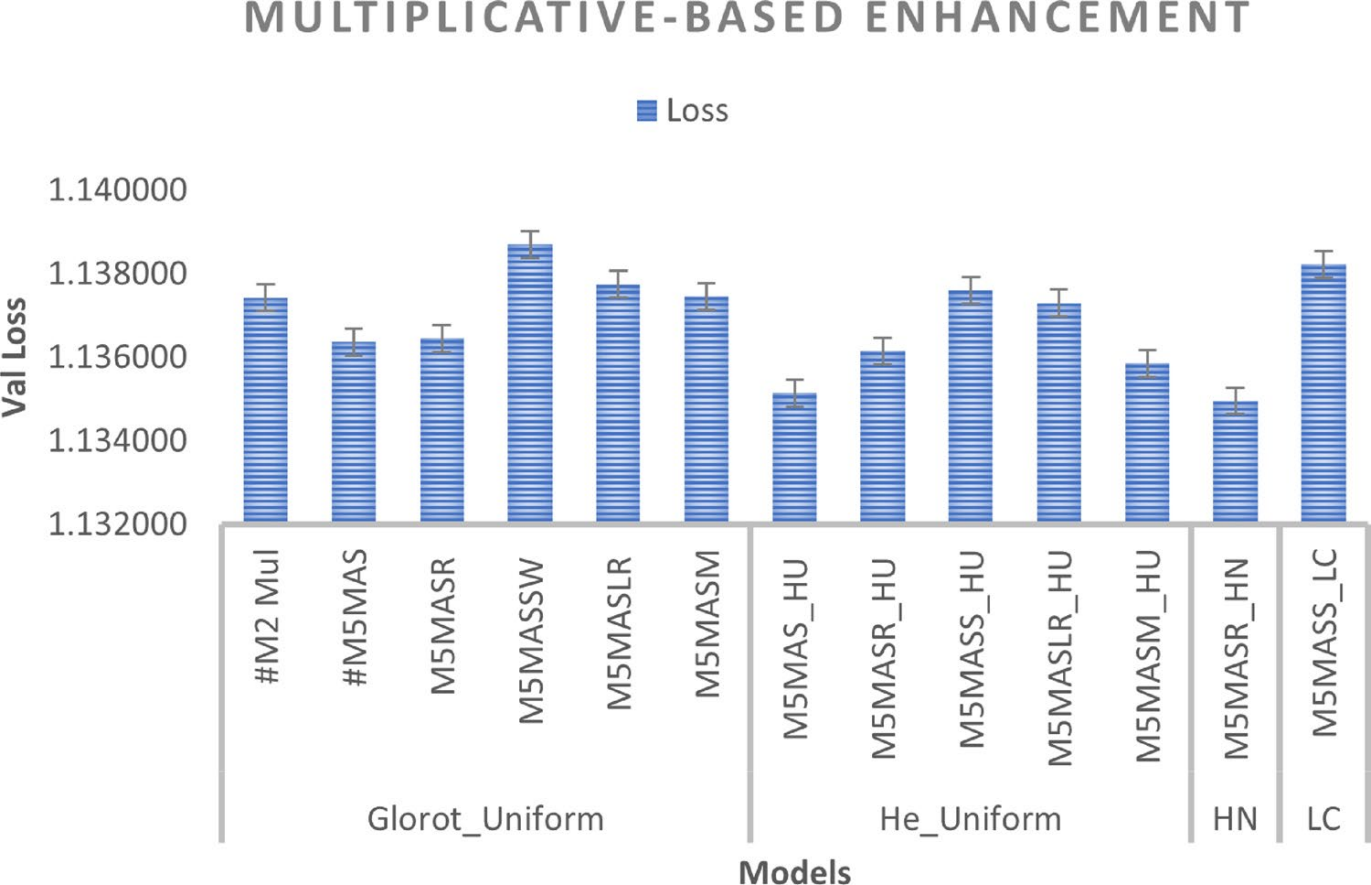
Multiplicative-based enhancement (performance during training phase).

**Fig. 10:**
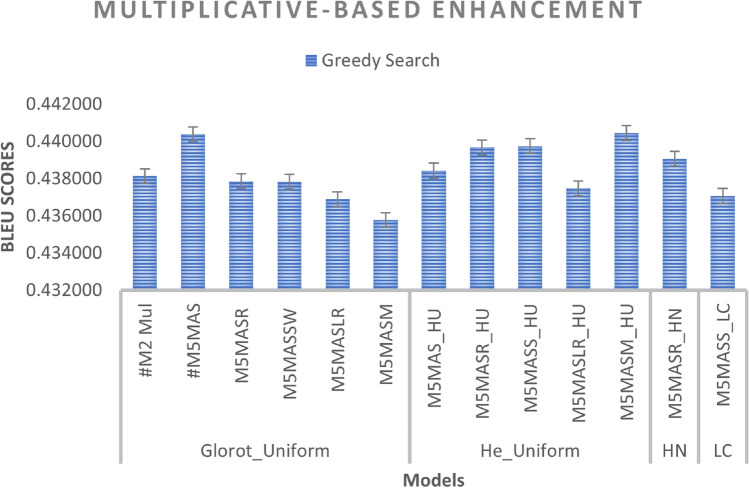
Multiplicative-based enhancement (performance during inference phase-Greedy search-based prediction Technique).

**Fig. 11:**
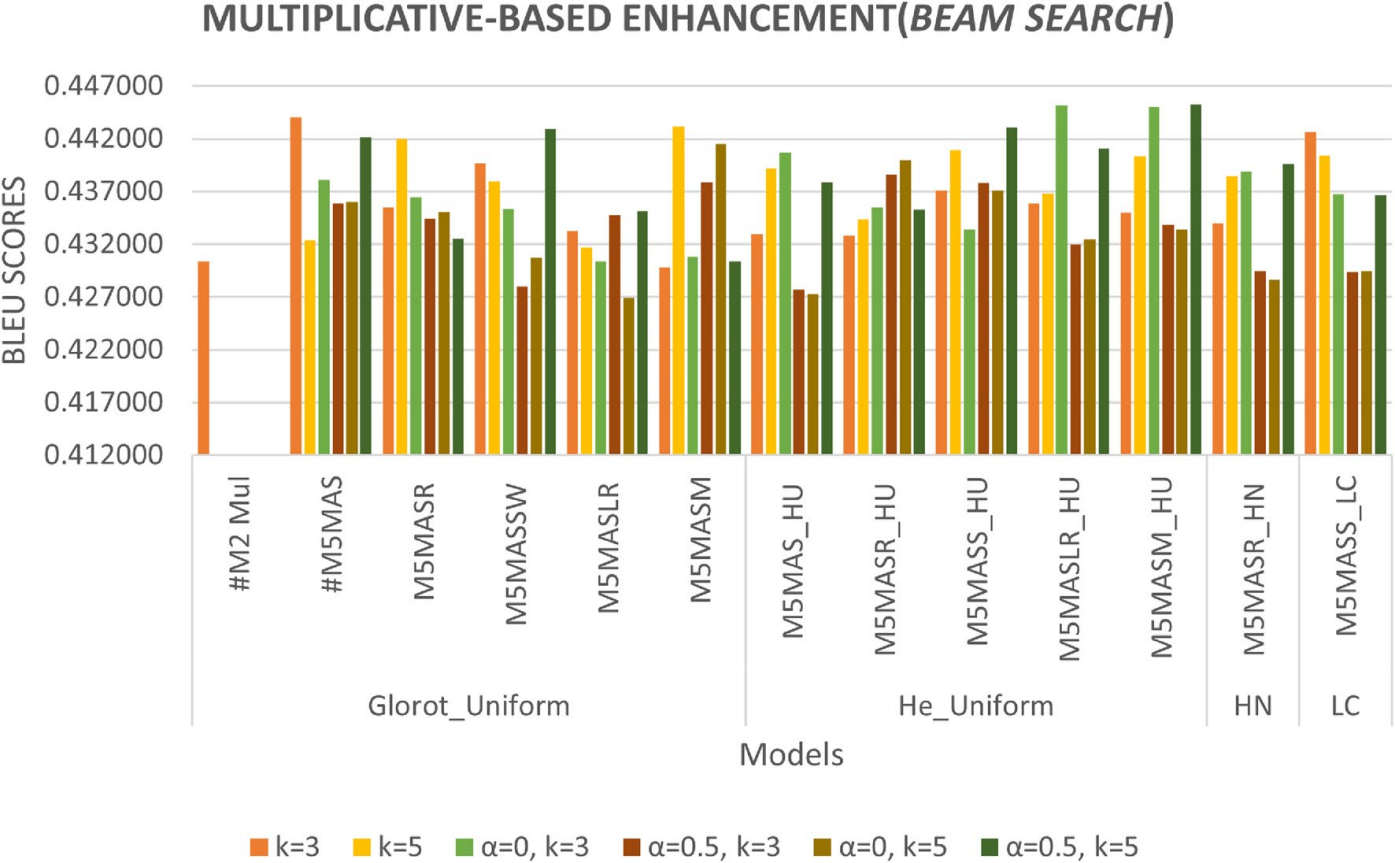
Figure 9 Multiplicative-based enhancement (performance during inference phase- Greedy search-based prediction technique).

We improved the base model by incorporating a non-linearity function with a scalar factor and combining both with different WI as well as activation functions. As can be seen in Figs. 10 and 11, the M5MASM_HU model achieves the best result in BLEU score performance for both greedy and beam search with parameter size 5 and penalty 0 (*k* =5, $\alpha$ = 0), while the M5MASR_HN model achieves the lower loss in training compared to #M2-Mul (base model), as shown in Fig. 9.

*Effect of WI*: We compared the standard weight initialized in Keras (Glorot_Uniform) with other WI, including He_Uniform, He_Normal, and Le_Cun. Across all experiments, 57 per cent of the models performed better with the weight initializer He_Uniform, suggesting that this weight initializer is a viable option for future studies.

*Effect of activation functions*: We compared the default activation (tanh) used in Keras with other activation functions such as ReLU, Swish, Leaky_ReLU, Mish, and SELU, as shown in Fig. 10. It can be seen that the activation functions tanh, ReLU and Mish regularly give better results since the frequency of occurrence achieving promising results among them is the same. Therefore, we can opt for these activations as the activation function in the multiplicative-based model. However, Mish with the He_Uniform initializer gives the best results among the proposed extension models and can possibly be used in future implementation.

*Effect of beam search parameters*: In the inference phase, we tested different beam search parameters in terms of size (*k*) and penalty ($\alpha$) and examined the effects of these parameters on the proposed models. As can be seen in Fig. 11, the parameters beam size *k*=5 and penalty $\alpha$=0.5 were used most frequently, with 41.67 per cent in the tested number of experiments, and provided promising results among the different models (M5MASSW, M5MASLR, M5MASS_HU, M5MASM_HU and M5MASR_HN). Meanwhile, this parameter achieves the best results when the initializer He_Uniform is used with the activation function Mish, indicating that it is promising for future experiments.

#### Comparisons of the best models among the different proposed model extensions against the base model

A comparison was made between the base model with the additive and multiplicative-based model selection. This selection is based on the performance of the model which has a lower loss and a high BLEU score. Thus, nine models were compared, of which five are additive and the rest are multiplicative, as depicted in Table 13.

**Table 13:** A comparison of proposed extension models (additive and multiplicative-based ASF) with the baseline models.

Model	Loss	Val loss	BLEU						
			Greedy search	Beam search					
				k = 3	k = 5	$\alpha$ = 0, k = 3	$\alpha$ = 0.5, k = 3	$\alpha$ = 0, k = 5	$\alpha$ = 0.5, k = 5
#M3-Add	1.073800	**1.138623**	**0.438506**	**0.440251**					
#M2-Mul	1.071038	1.137435	0.438132	0.430391					
M4ALR	1.073854	1.137080	0.438971	0.432181	0.436614	0.433565	0.434902	**0.436234**	0.431222
M4AS	1.071599	1.135726	0.437864	0.428708	0.435355	0.446262	0.430031	0.434155	0.433872
M4AHR	1.070105	1.135668	0.439154	**0.451428**	**0.442181**	0.437486	0.437808	0.433220	0.440940
M4AHM	1.070243	**1.134345**	0.439197	0.441446	0.436521	0.438819	0.427420	0.432288	0.440766
M4ALS	1.070615	1.136054	**0.440779**	0.438271	0.438186	**0.459270**	0.440892	0.433620	0.442607
M5MAS	1.071383	1.136370	0.440390	0.444001	0.432404	0.438130	0.435834	0.436026	0.442150
M5MAS_HU	1.070049	1.135154	0.438430	0.432948	0.439174	0.440727	0.427681	0.427286	0.437871
M5MASM_HU	1.070757	1.135852	0.440445	0.435013	0.440360	0.445040	0.433824	0.433408	**0.445236**
M5MASR_HN	1.069998	1.134963	0.439069	0.433964	0.438484	0.438871	0.429469	0.428625	0.439606

Significant values are in [bold].

**Fig. 12:**
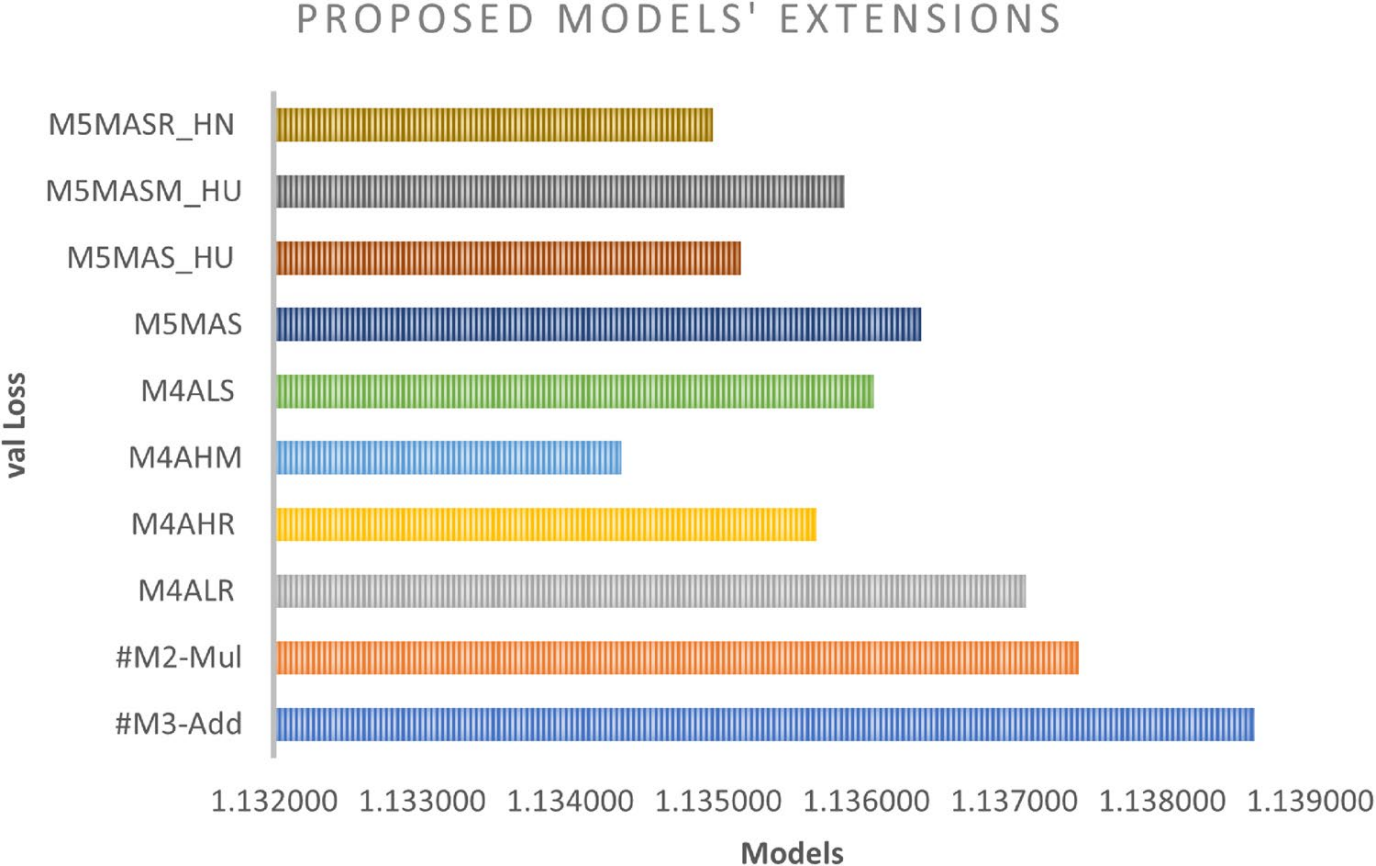
Comparison between the best of the proposed models’ extension based on the loss during the training phase.

**Fig. 13:**
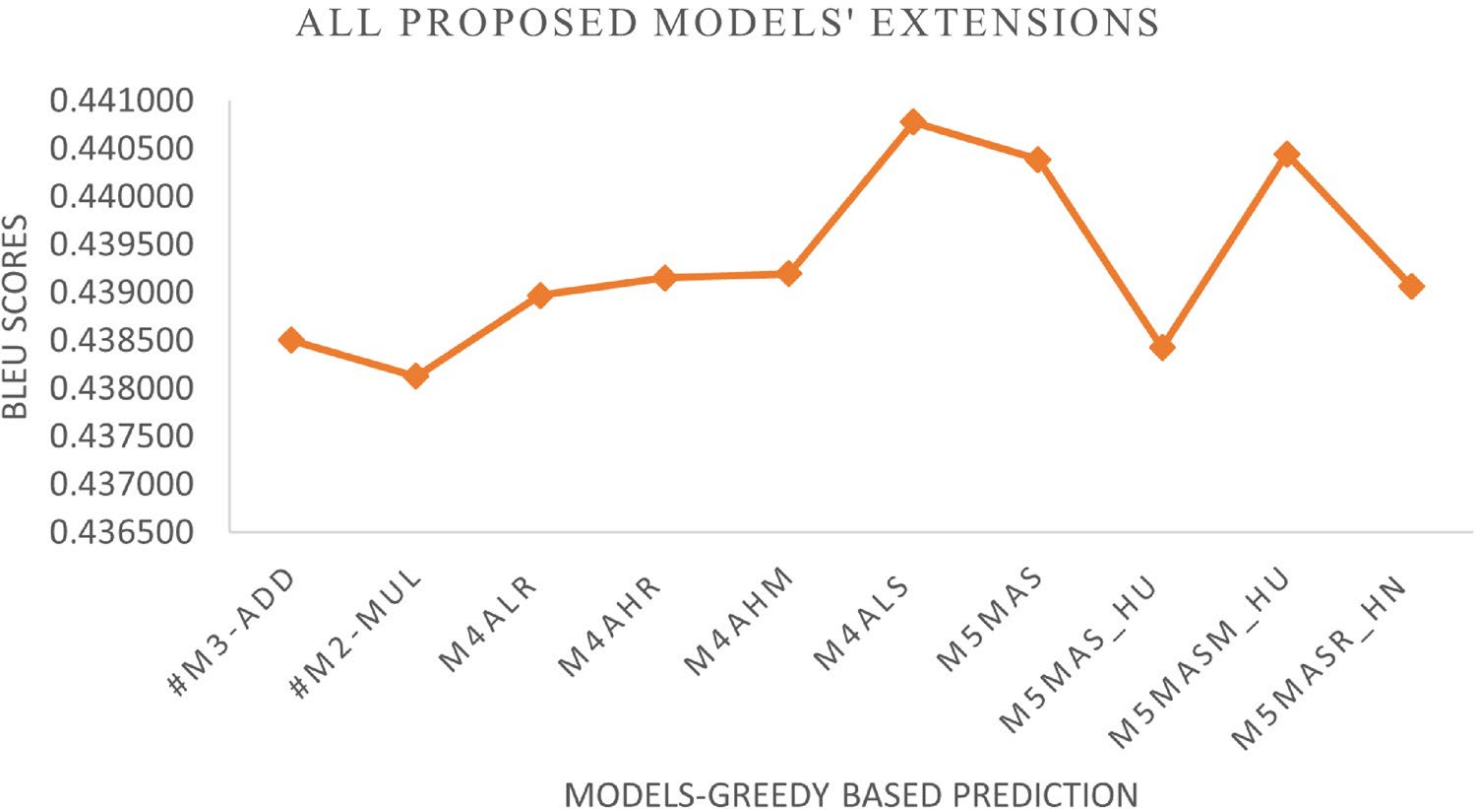
Comparison between the best of the proposed models’ extension in greedy-based prediction during the inference phase.

**Fig. 14:**
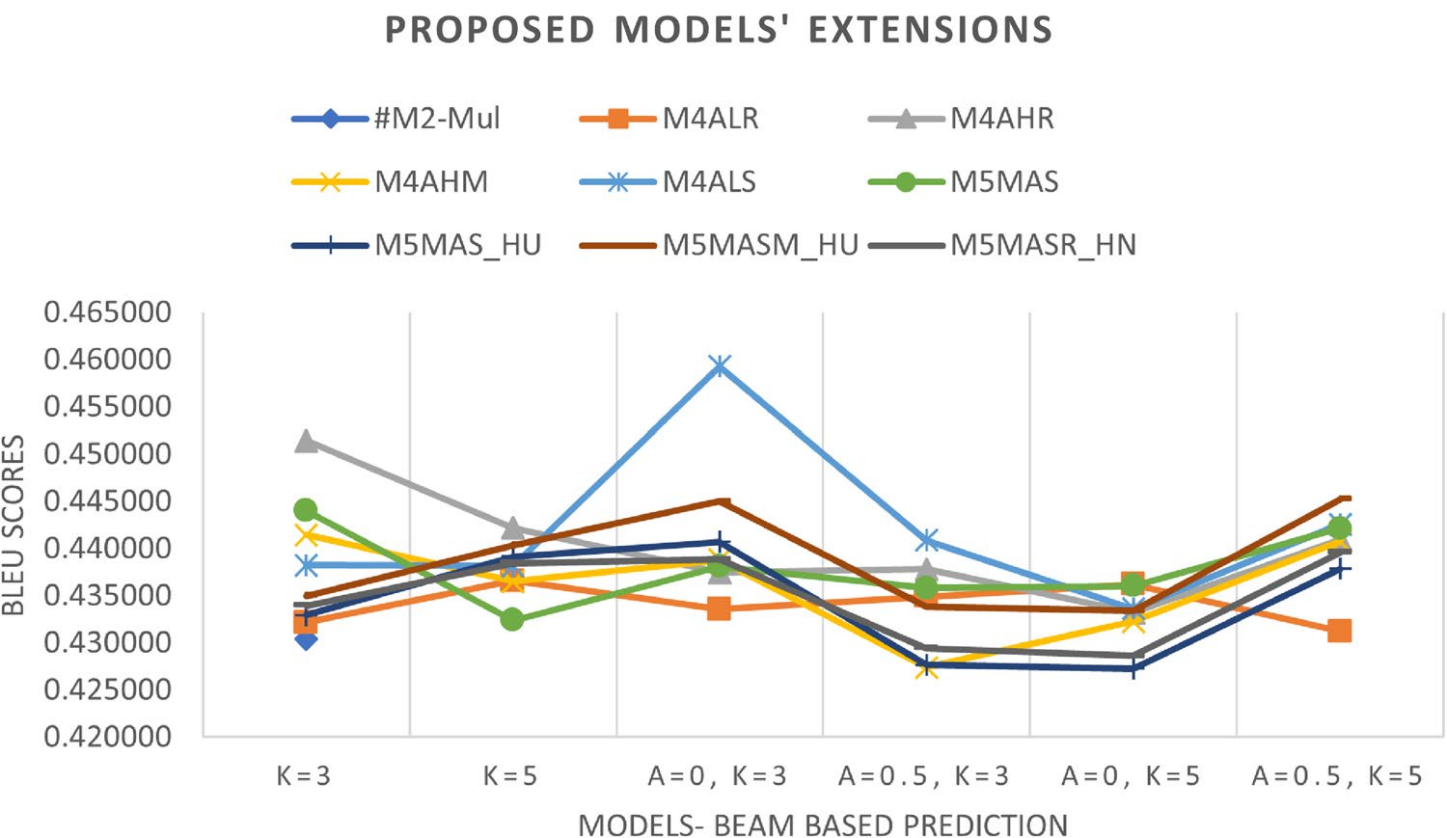
Comparison between the best of the proposed models’ extension in beam search-based prediction during the inference phase.

The results of the experiments as shown in Table 13 and Figs. 12, 13 and 14, confirm the fact that the extensions of the proposed models are almost an improvement. It can be concluded that the inclusion of the scaling function and the extension with different weight initializers and activation functions provide a significant improvement compared to the mere use of the vanilla attention and thus have an impact on the model performance. Moreover, the improvement of the additive-based ASF models dominated over the multiplicative-based models in almost all cases of the conducted experiment. This result indicates that the additive-based ASF has a greater impact on capturing and aligning the relevant information between the query and response pair of the dataset than the multiplicative-based one.

Comparing different weight initializers Glorot Uniform and He_Uniform, it is found that the He_Uniform initializer performs better in most cases, indicating the stability of this initializer in distributing the weights among the models. However, the combination of the Le_Cun initializer and the SELU activation function performs better in prediction than Glorot and He_Uniform in most cases. For beam-based prediction, as seen in Fig. 14, most models perform better with a beam parameter of size *k*=3 and a penalty of $\alpha$=0. However, the performance of the other beam parameters is comparable for most models and difficult to interpret because the beam size parameter tested for this experiment is small (width). A higher beam parameter usually results in better response prediction quality, but would consume a lot of memory and computational power compared to a small beam size. Due to this limitation, we could only run 1,000 samples of the validation set to investigate the beam search technique with and without the penalty assumption. Although a small parameter was used in this experiment, the results show that applying a penalty to the beam search achieves better prediction performance, indicating a promising and valuable technique for future studies. To facilitate interpretation, Table 13 provides a structured overview of BLEU performance under different beam configurations. The proposed models, especially M4ALS and M4AHM, consistently outperform the baseline models across all tested combinations of beam size $k \in \{3, 5\}$ and penalties $\alpha \in \{0, 0.5\}$. Notably, these models achieve their highest BLEU scores with $k=3$ and $\alpha =0.5$, suggesting that moderate penalization paired with narrow beam width may suffice for strong prediction alignment in customer service tasks. Despite the limited beam width due to computational constraints, the observed pattern underscores the robustness of the proposed architecture and decoding strategy. This structured analysis also complements the qualitative findings discussed later in section “Qualitative analysis”.

Another essential measure to ensure the performance of the proposed models is the use of a quantitative approach called the significance test. The significance test used in this study, following the work of^37^ is Welch’s t-test. The Welch’s t-test is a statistical hypothesis test performed at a 5 per cent significance level (0.05) to test whether the improvement in our model is significant. This is done to show that the best accuracy (BLEU score) achieved by our proposed models is statistically significant and not due to chance or random factors. From Table 14, it can be seen that our proposed scaling factors combined with the deep learning neural model environment significantly outperformed the baseline models, and the improvement is statistically significant at *p* < 0.05. This shows the effectiveness of our proposed models in generating response predictions compared to the baseline models.

**Table 14:** Selective model statistical significance test based on Welch’s t-test.

Models	BLEU scores									
	#M2		Welch’s two samples t-test			#M3		Welch’s two samples t-test		
	Mean	SD	df	t	p	Mean	SD	df	t	p
	0.388942	0.203934	–	–	–	0.395229	0.201954	–	–	–
M4ALR	0.437864	0.200670	355603.433	– 72.110	< 0.001	0.437864	0.200670	355681.522	– 63.154	< 0.001
M4AHR	0.400632	0.197236	355300.012	– 17.376	< 0.001	0.400632	0.197236	355497.382	– 8.072	< 0.001
M4AHM	0.437197	0.200865	355614.266	– 71.094	< 0.001	0.437197	0.200865	355685.605	– 62.137	< 0.001
M4ALS	0.440779	0.201355	355638.412	– 76.279	< 0.001	0.440779	0.201355	355692.862	– 67.358	< 0.001
#M5MAS	0.440390	0.200503	355593.630	– 75.864	< 0.001	0.440390	0.200503	355677.494	– 66.924	< 0.001
M5MAS_HU	0.438430	0.200958	355619.172	– 72.893	< 0.001	0.438430	0.200958	355687.306	– 63.947	< 0.001
M5MASM_HU	0.440445	0.200923	355617.311	– 75.868	< 0.001	0.440445	0.200923	355686.673	– 66.937	< 0.001
M5MASR_HN	0.439069	0.202519	355678.754	– 73.553	< 0.001	0.439069	0.202519	355693.227	– 64.643	< 0.001

### Limitations and future works

While the proposed lightweight neural attention-based model introduces significant improvements in efficiency and scalability for seq2seq architectures, several key limitations remain. Addressing these challenges will be crucial for ensuring its broader applicability in real-world service chatbot environments and beyond.

The model has been evaluated in a controlled offline setting using benchmark datasets. However, its performance in real-time applications remains untested. Factors such as inference speed, latency, and adaptability to dynamic user interactions have yet to be analyzed. Future research should focus on deploying the model in practical chatbot environments to assess its robustness under real-world conditions, where computational constraints and network variability may impact performance.

The model’s lightweight design aims to reduce computational overhead while maintaining response quality. However, balancing efficiency and contextual accuracy remains a challenge. While the proposed approach enhances seq2seq architectures, it has not been benchmarked against state-of-the-art transformer-based models, such as GPT-4 or T5, which may provide superior contextual understanding but at a higher computational cost.

Although smaller transformer variants such as TinyBERT and DistilBERT exist ^38,39^, they were not considered in this study for several reasons. First, such models often rely on extensive pre-training with general corpora, which limits their adaptability to domain-specific customer service data without substantial fine-tuning ^40^. Second, their black-box nature can hinder interpretability, especially when compared to our scalar-attention mechanism, which provides more transparent control over alignment and attention distribution. Finally, fine-tuning even small transformers requires careful hyperparameter optimization and considerable GPU memory, which is beyond the scope and computational cost of this study. Future studies should consider benchmarking against these lightweight transformer variants and investigate hybrid approaches that integrate elements of both seq2seq and transformer architectures to achieve an optimal trade-off between computational efficiency and response quality.

The model’s performance has been validated within the domain of service chatbots, yet its adaptability to other NLP tasks, such as machine translation, text summarization, and dialogue-based recommender systems, remains unexplored. Future research should assess whether these enhancements generalize effectively across diverse sequence prediction tasks. This could involve integrating the scalar attention mechanism into widely adopted architectures such as transformer-based models, to evaluate whether similar improvements in alignment efficiency and interpretability can be achieved across diverse NLP tasks.

This study demonstrates improvements over the seq2seq baseline models, yet no direct performance comparison with cutting-edge models, such as transformer-based conversational agents, has been conducted. The increasing dominance of large-scale pre-trained models highlights the need for comparative evaluations that measure the strengths and limitations of the proposed models in relation to the latest advances in deep learning for NLP. Such comparisons will provide deeper insight into its competitiveness and potential for further enhancement.

The proposed modifications introduce structural enhancements to the attention mechanism, yet their effectiveness may be highly dependent on hyperparameter configurations such as weight initialization, sequence length constraints, and learning rate adjustments. While preliminary experiments indicate performance gains, further systematic optimization and fine-tuning, including evaluation in *MultiWOZ 2.4*, *PersonaChat*, and the *Ubuntu Dialogue Corpus*, are needed to improve the model’s generalization across different datasets and application scenarios. By addressing these limitations and exploring new optimization strategies, the proposed model can be further refined to serve as a scalable and efficient solution for service chatbots and other NLP applications.

Lastly, this study primarily adopts BLEU score and validation loss as the core evaluation metrics, given their widespread use and acceptance in sequence modeling and chatbot research ^5,20,41^. BLEU, originally developed for machine translation, remains a practical benchmark in dialogue systems, offering an interpretable measure of response quality through n-gram overlap ^42^. Its focus on precision and linguistic alignment makes it particularly suitable for evaluating on-topic, coherent responses in customer service chatbots. While alternative metrics such as ROUGE and METEOR offer valuable insights, particularly in tasks involving summarization and paraphrase generation, their focus on recall and synonym matching is less aligned with the primary objectives of this study. For this reason, BLEU was considered more appropriate for evaluating the effectiveness of the proposed attention mechanism. Nonetheless, we recognize the limitations of relying exclusively on BLEU ^43^. Future work will therefore investigate the inclusion of complementary metrics, including ROUGE, METEOR, and emerging semantic evaluation methods based on large language models (LLMs), to enable a more comprehensive assessment of response quality, contextual relevance, and semantic appropriateness.

### Qualitative analysis

**Fig. 15:**
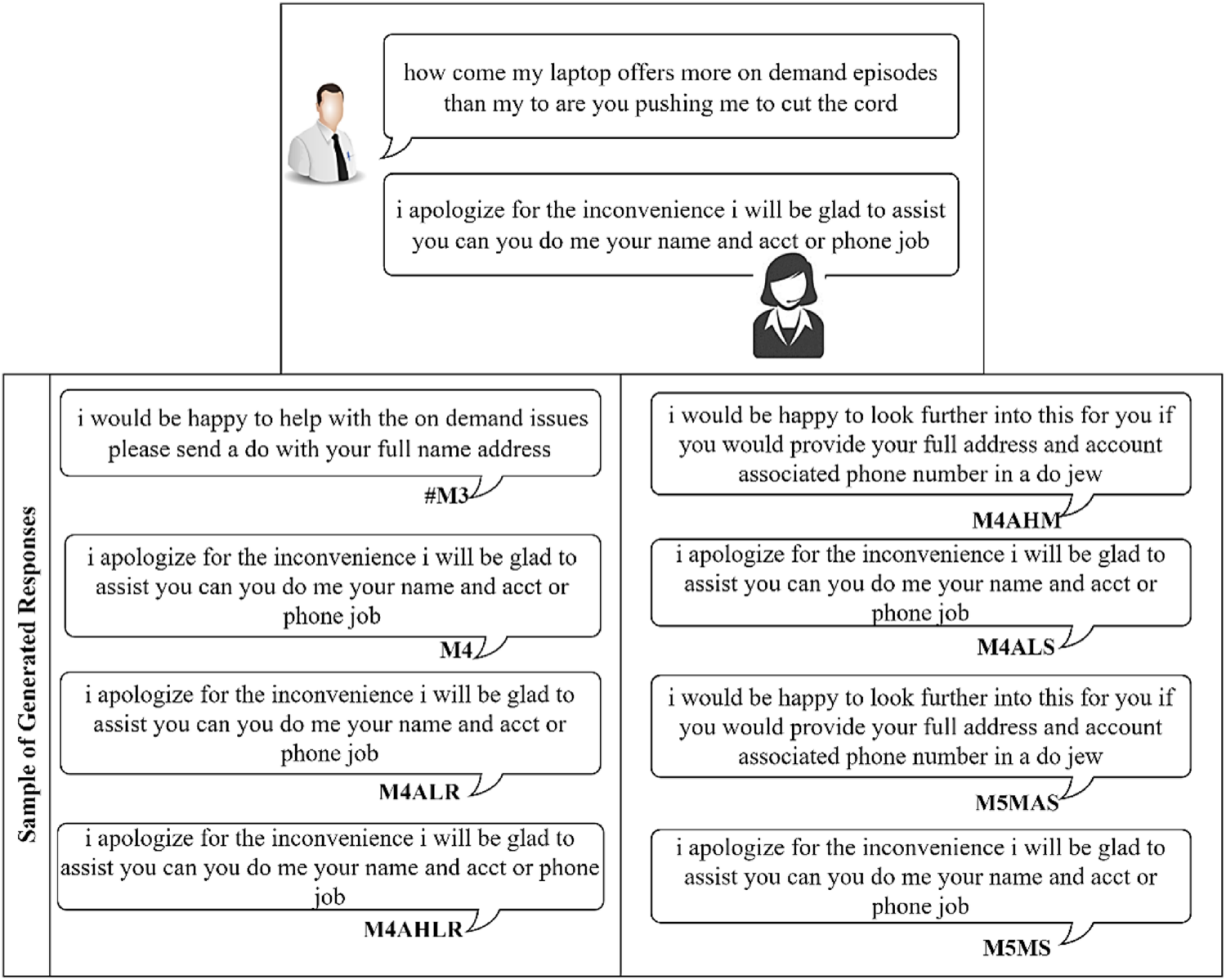
Sample of good-generated response by variants of models.

**Fig. 16:**
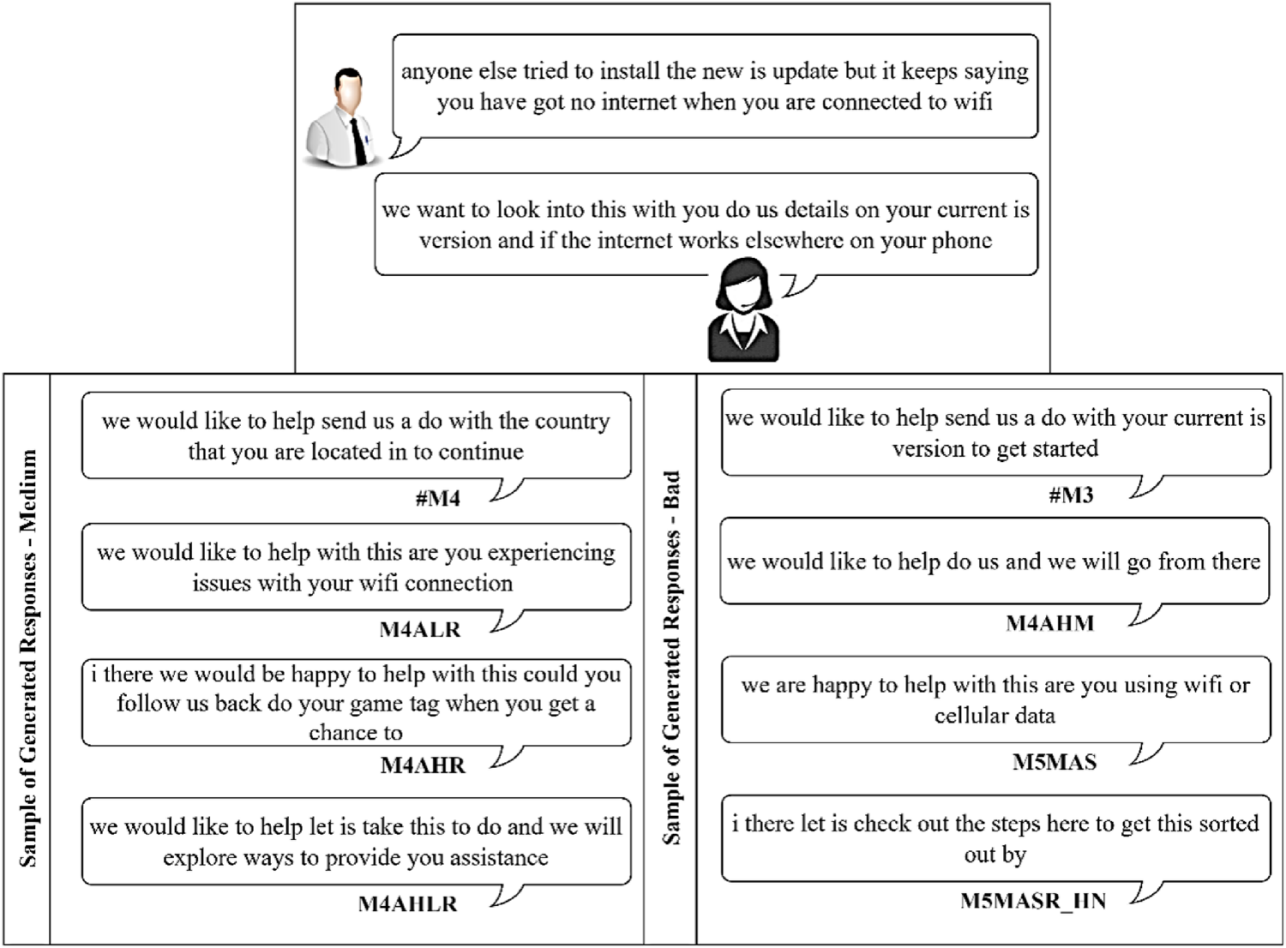
Sample of medium and bad-generated response by variants of models.

To qualitatively comprehend the performance of the model created with a different experiment configuration, this study provides an example of automatically generated responses to a specific customer query. Two types of generated response forms for the chatbot should be able to respond with an accurate answer: single-turn and multi-turn. The single-turn response form indicates that chatbots tend to respond to a single text without remembering any information from the conversation prior to that point. In contrast, the multi-turn response form shows the chatbot’s ability to remember information from previous conversations, and this knowledge helps it answer questions related to previous contexts. Below are examples of the single-turn responses generated by all the models, i.e., examples of good results (see Fig. 15) and examples of medium and poor results (see Fig. 16). For good results, some of the models may give actual human-generated responses where the BLEU score is one.

We also extended qualitative analysis of chatbot-generated responses to specific customer queries, which can be based on informative and emotional questions. For the informative customer query *“how am i supposed to order the terry chocolate orange a flurry on if its not on the user menu?”*, the predicted responses are shown in Table 15. In contrast, Table 16 shows an example of an emotional question. As observed, most neural models can provide surprisingly accurate answers when predicting responses, especially the proposed models compared to the baseline for this example question. This may happen as the model puts more weight on the word ‘help’ when predicting the answer since the actual answer is not very specific, and, therefore, it is able to make an accurate prediction. However, if the question is too specific, the model may have difficulty predicting the answer, resulting in a less accurate answer that is unrelated to the question. In addition to the informative questions, the models might focus on the word *‘sorry’* as all models detected this word in their answer prediction, although different answers were given, but were reasonable, except for the model M5MAS_HU. Thus, it is evident that the models have a sense of emotion and can predict emotional responses during a conversation, which could be beneficial for customer service. Compared to the baseline models presented previously, the proposed model for single turn noticeably improved the response prediction; it was observed that most of the proposed models could predict the response as the actual response compared to the baseline model, indicating that the proposed model improves response prediction during conversation.

**Fig. 17:**
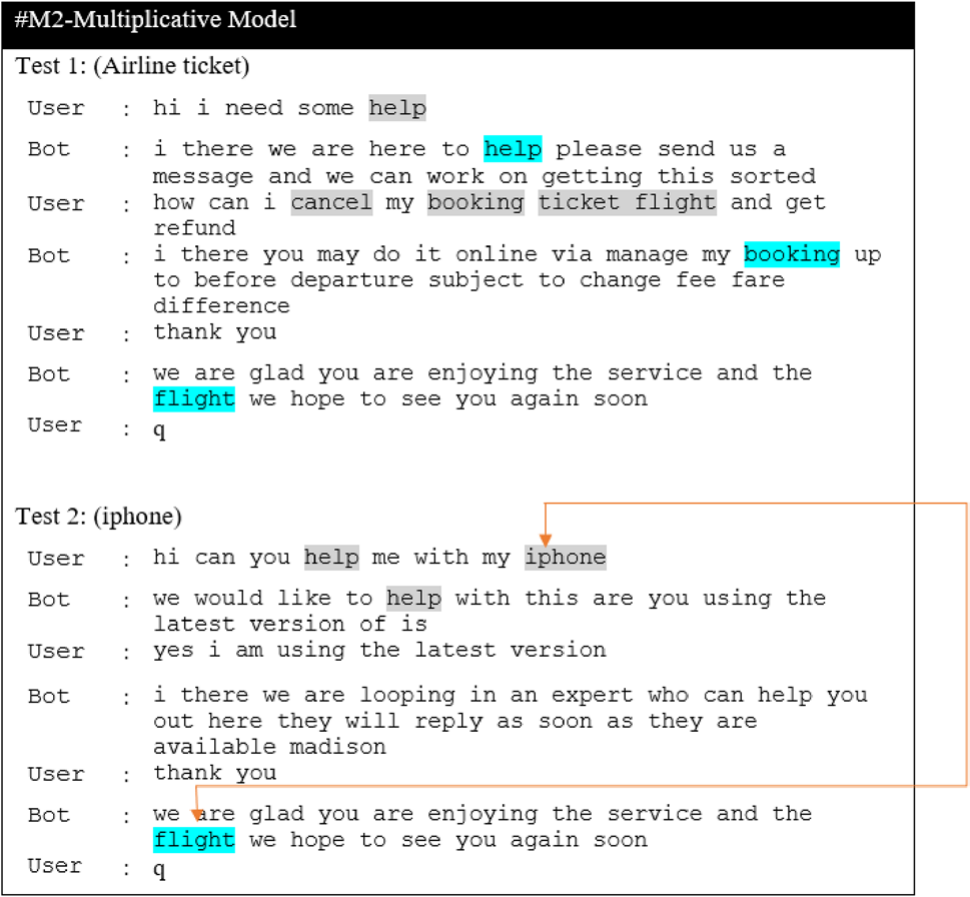
Samples of multi-turns types generated responses from #M2-Multiplicative Model.

To complement this, we conducted an error analysis to align qualitative shortcomings with BLEU scores. While BLEU scores offer a broad quantitative metric for model quality, our qualitative analysis reveals specific response patterns that align closely with these metrics. For example, models such as #M3, M4AHM, MSMAS and MSMASR_HN in Fig. 16 provided a sample of weak response. These responses were either too general (e.g., “we would like to help do us...”) or semantically incoherent (e.g., “there let is check out...”), indicating that the models failed to capture important contextual cues. Moreover, in multi-turn interactions, such models struggled to maintain conversational history or recall earlier user intents, for instance, in Test 2, where model #M2 failed to address the *iPhone* query appropriately (see Fig. 17). These errors, such as repetitive phrasing, missing entity references, and default template replies, consistently aligned with lower BLEU scores. This observation supports our claim that BLEU scores correlate with real-world response quality and conversational coherence.

**Table 15:** Example of generated response for informative type question.

Customer questions	
	how am i supposed to order the terry chocolate orange a flurry on if its not on the user menu
#M2	we are sorry to hear this please do us your contact info and store address thanks
#M3	we can take a look send us a note via so our team can get in touch
#M4	we are here to help send us a do with your email address so we can assist
M4ALR	we are here to help can you please send us a do with your email address so we can connect
M4AHR	we are here to help can you please send us a do with your email address so we can connect
M4AHM	i there we are here to help can you please send us a do with your email address so we can connect
M4ALS	we are here to help can you please send us a do with your email address so we can connect
M5MAS	we are here to help can you please send us a do with your email address so we can connect
M5MAS_HU	we are here to help can you please send us a do with your email address so we can connect
M5MASM_HU	we are here to help can you please send us a do with your email address so we can connect
M5MASR_HN	we are here to help can you please send us a do with your email address so we can connect
**Actual response**	**we are here to help can you please send ** **us a do with your email address so we** ** can connect**

**Table 16:** Example of generated response for emotive type question.

Customer questions	poor amazon india delivery no communication for not delivering amazon india bad customer experience
#M2	i am sorry for the trouble with the delivery of your order please report this to our support team here and we will check this in
#M3	am sorry for the unpleasant experience please reach out to us here and we will help you with this
#M4	we are sorry for any discomfort and we will be sure to pass your comments on to our catering team
M4ALR	sorry for the hassle please report this to our support team here and we will check this
M4AHR	sorry for the trouble you are facing with delivery let us look into it
M4AHM	we are sorry for the frustration and we are working on making our team to get you to your destination we will have your bags out as soon as possible
M4ALS	sorry for the hassle please report this to our support team here and we will check this in
M5MAS	sorry for the trouble with the delivery of your order we certainly did not expect this to happen is
M5MAS_HU	we are always looking to expand our military to provide more information and safety reasons no
M5MASM_HU	we are sorry for the wait we will have you wheel is up and on your way
M5MASR_HN	sorry for the unpleasant experience please report this to our support team here and we will check this
Actual response	**sorry for the hassle please report this to** ** our support team here and we will ** **check this in**

**Fig. 18:**
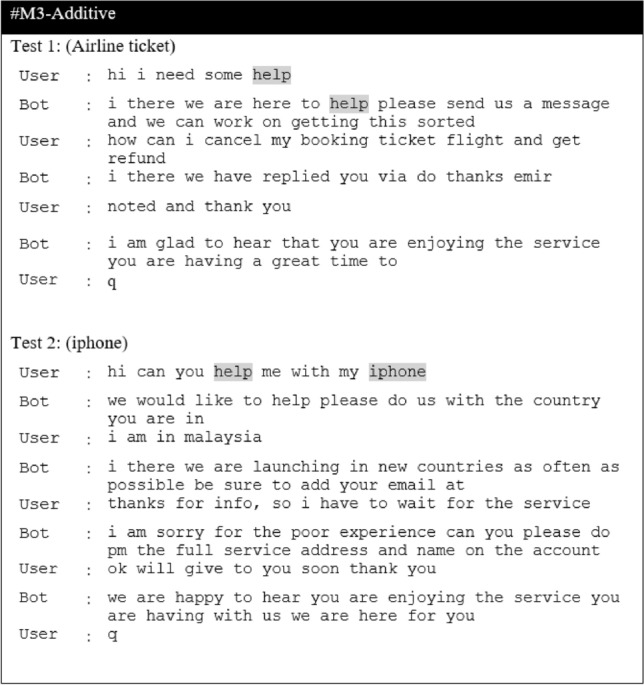
Samples of multi-turns types generated responses from #M3-Additive Model.

**Fig. 19:**
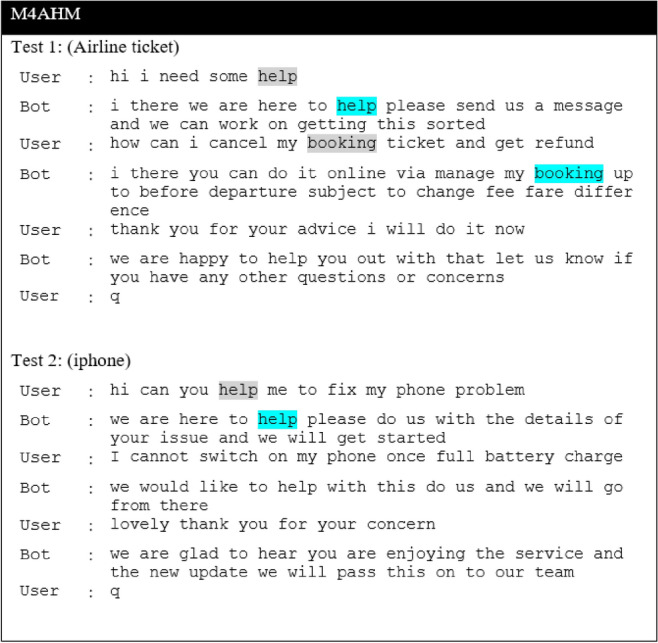
Samples of multi-turns types generated responses from M4AHM.

**Fig. 20:**
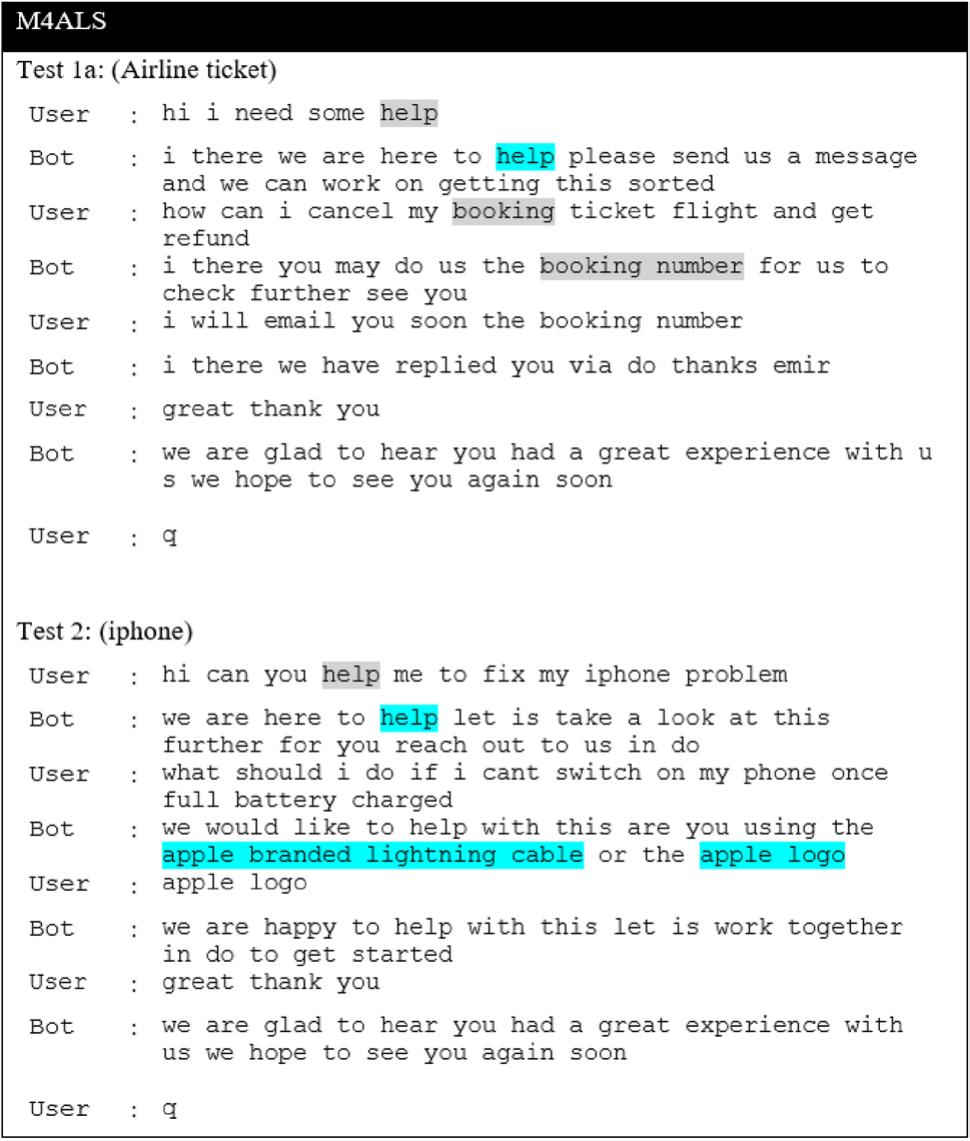
Samples of multi-turns types generated responses from M4ALS.

Apart from the single-turn conversation types, see Figs.  17, 18, 19 and 20 for examples of multi-turn responses generated by the neural models. The multi-turn chatbot response is queried based on the frequency of occurrence of words in the dataset, e.g. *‘help’*, *‘phone’*, *‘service’*, etc. Figure 17, 18, 19 and 20 shows an example of a conversation between the user and the chatbot over different selected models. We tested both the base model and the improved model for the purpose of quality analysis. These figures also show two examples of questions users asked the models related to customer service, namely airline tickets and iPhone, to determine the models’ ability to predict an accurate answer based on the questions asked.

Four selected models were analyzed, namely the basic #M2 multiplicative and #M3 additive models, as well as the proposed M4AHM and M4ALS models. In general, it is quite difficult to analyze and interpret the effectiveness of the models in response prediction, as the models were trained with only 10 epochs. The possible number of epochs is not sufficient to train the developed models. As can be seen from the model’s responses to the user’s utterances *‘help’*, *‘flight’*, *‘iphone’*, and *‘booking’* (highlighted in blue for the model/bot), the model can use its attention mechanism to recognize the context. In the first utterance of the user asking for help, most models recognize the word *‘help’* as the model focuses more on this word and proceeds to the next utterance to continue the conversation with the user. In addition, the models may also make suggestions and ask the user for details to solve the user’s problem. This shows that the models are able to stay with the conversation and remember the previous information without giving an irrelevant response, except for the #M2 Multiplicative model for test 2, which cannot recall the word *‘iphone’* at the end of the conversation with the user about the iPhone. The developed models can also provide appropriate responses and know how to end the conversation when the user wants to end the conversation topic. Overall, while all models provide a basic level of response to user queries, the proposed models (M4AHM and M4ALS) seem to perform better in terms of relevance and contextual understanding, knowing what to respond to based on the user’s utterances without providing overtly unrelated or inappropriate responses. They also seem to be more engaged in interacting with the user and provide a more personalized and contextualized service. Still, there are some areas where each model could improve, particularly in providing clearer and more concrete solutions to users’ problems.

## Conclusion

This paper presents a significant advancement in the field of neural generative seq2seq attention mechanisms, specifically for service chatbot applications. The proposed enhancements introduce a lightweight adaptation of the attention score function (ASF), allowing the model to focus more precisely on relevant input-output sequence information. This refinement results in responses that are not only more contextually appropriate but also computationally efficient. These efficiency gains are particularly valuable for real-world deployment scenarios, where minimizing latency and computational load are crucial for maintaining responsiveness in high-traffic or resource-limited environments. A key innovation of this study lies in modifying the attention structure by incorporating a scalar function, thereby improving both scalability and performance within deep learning architectures optimized for network training. Our investigation highlights the impact of integrating a scaling factor into the ASF, which mitigates gradient instability, improves attention alignment, and enhances chatbot response accuracy. Through systematic experimentation with different activation functions and weight initializers, our results demonstrate that these structural enhancements significantly improve model performance. The findings reveal that additive-based ASF consistently outperforms those using multiplicative-based models, highlighting their superior ability to capture and align relevant information within query-response pairs. Moreover, our study found that the $He\_Uniform$ weight initializer generally outperforms the $Glorot\_Uniform$ initializer, indicating its greater stability in weight distribution among models. Notably, the combination of the $Le\_Cun$ initializer with the *SELU* activation function has consistently outperformed both the *Glorot* and $He\_Uniform$ initializers, marking a promising direction for further optimization. From an inference efficiency perspective, our findings indicate that beam search decoding with specific configurations *k*=3 and $\alpha$=0 enhances response prediction performance. These findings highlight the importance of parameter selection in balancing computational cost and response accuracy. While this study employs minimal parameters, the demonstrated performance gains suggest the potential for further optimizations through adaptive decoding strategies. Despite these advancements, challenges remain regarding the model’s scalability and real-time deployment, particularly in handling dynamic and large-scale conversational datasets. While these limitations have been acknowledged, they also present opportunities for further research. Future work should explore integrating hybrid architectures that balance computational efficiency and accuracy, such as sparse attention mechanisms and transformer-based refinements. Moreover, expanding this approach beyond chatbots to other sequence prediction tasks, including recommender systems, sentiment analysis, and machine translation, could further validate its adaptability and broader impact. Beyond chatbot systems, the proposed mechanism could be extended to dialogue-based tutoring platforms, conversational healthcare assistants, and other real-time interactive systems where contextual relevance and inference efficiency are critical. By refining attention mechanisms within seq2seq models, this research contributes to the ongoing evolution of AI-driven conversational agents. The insights gained from this study not only advance chatbot design but also serve as a foundation for future innovations in scalable and efficient neural attention mechanisms.

## Figures and Tables

**Fig. 21:**
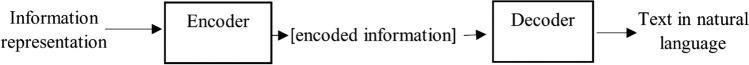
Diagram of an encoder-decoder architecture for NLG.

**Algorithm 2:**
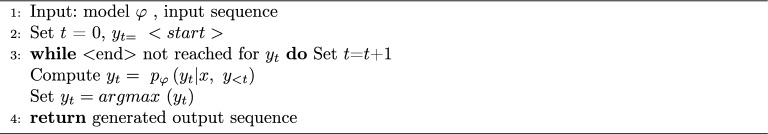
Greedy search decoding

**Algorithm 3:**
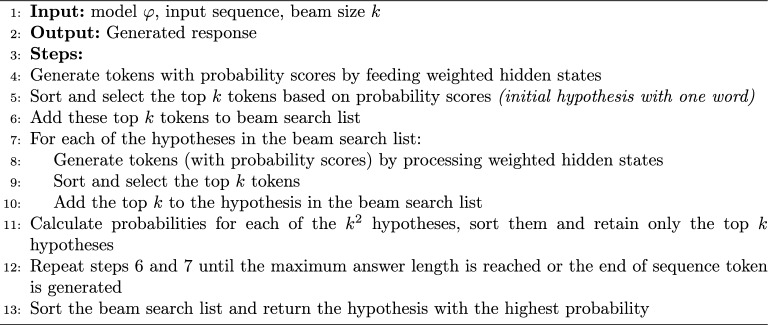
Beam Search decoding.

## Data Availability

The dataset used and analysed in the current study, *Customer Support on Twitter (CST)*, is publicly available in the Kaggle repository and can be accessed at https://www.kaggle.com/datasets/thoughtvector/customer-support-on-twitter.

## Appendix A

Seq2Seq learning task model, introduced in 2014 by Sutskever et al.^10^, serves as a cornerstone for numerous natural language processing tasks, including machine translation and chatbot development. This learning model, an innovative adaptation of recurrent neural networks (RNNs), effectively handles seq2seq modeling problems arising from variable-length input and output. This adaptability has proven critical in complex tasks such as machine translation and chatbot development, where traditional RNNs falter due to the variable input and output length. The most commonly used architecture for constructing seq2seq learning tasks is known as the encoder-decoder architecture. Seq2seq models can be adapted to convert an abstract representation into a natural language^44,45^, as depicted in Fig. 21.

The seq2seq learning task involves an encoder-decoder architecture that comprises three components: encoder, context vector (final hidden/internal state vector), and decoder. It can be extended with an additional layer, the attention layer, to improve prediction, as described in the following subsections.

### Encoder architecture

The purpose of the encoder is to convert the input sentence into a defined (fixed-size) hidden representation, effectively conveying the information. The decoder on its part transforms this hidden representation into output. At its core, this model is composed of two RNNs. RNNs can help overcome classical neural networks’ limitations by assuming that inputs are interdependent and use sequential information. RNNs are designed in such a way that the network has a memory so that the information, which is sequential data, can be stored for understanding previous information through memorization. Therefore, RNNs are commonly referred to as ”recurrent” due to their ability to carry out the same operation on each element in a sequence, with the output being influenced by past calculations; moreover, they have a memory that preserves the previously computed information. This method can increase the network’s ability to handle meaning sequences. However, RNNs have short-term memory – they can quickly fail to remember what is seen in longer sequences. To alleviate this problem, other types of RNN cells have been introduced, namely Long Short-Term Memory (LSTM) and Gated Recurrent Unit (GRU), which provide better performance in learning long dependencies. Therefore, in this study, we use LSTM cells as the network structures of this architecture.

During the process where one RNN works as an encoder, it takes into account the context and most important parts of a given sequence. Another RNN serves as a decoder and uses this vector to produce the output sequence. As shown in Fig. 1, $x=\left\{ x_1,\ x_2,x_3,\ldots ..x_m\right\}$ represents the words contained in each input statement or utterance (where *m* is the length of the statement), which are mapped in the form of an embedded representation $\varphi (x_i)$. In this study, we used FastText pre-trained word embedding (as mentioned previously), where the input sequences are padded to a fixed length. In this study, we used FastText pre-trained word embedding (as mentioned previously), which captures semantic relationships between words by considering subword information. This allows the model to handle words that are out of vocabulary and improves overall performance. The input sequences are padded to a fixed length to maintain consistent input dimensions for the neural network. The masking parameter resulting from this padding is passed to the embedding layer so that the embedding layer ignores the masked tokens. The output of the embedding layer is passed to the LSTM layer. The LSTM layer takes an initial hidden state of the encoder as input. It manages all these embeddings and generates the hidden representation (or state) with the output vectors sequentially at each timestep. Thus, it takes a word with a hidden state of the previous state as input and returns an output, then it updates the hidden state until it reaches the end of the input token, specified as <EOS>.

However, in vanilla seq2seq architecture, the outputs at each timestep of the encoder part are all discarded because they are summarized by the context vector (*c*). This context vector attempts to encapsulate the information for all input elements so that the decoder can make an accurate prediction. Equations (1, 2) show how the hidden states and the context vectors are computed, respectively.

20 $$\begin{aligned} {\ h}_m&= f_1\left( \varphi (x_i),\ h_{m-1}\right) \end{aligned}$$

21 $$\begin{aligned} c\ &= f_2\left( \left\{ h_0,\ h_{1,\ }.\ .\ .\ h_M\right\} \right) \end{aligned}$$

Here, *h* represents the hidden state, *c* denotes the context vector derived from the encoder’s hidden state, and $f_1$, $f_2$ are nonlinear functions such as LSTM/GRU in this context. The context vector also functions as an initial hidden state of the decoder, facilitating the transfer of information from the encoder to the decoder. However, before transferring the information to the decoder, it is passed through bidirectional encoders with a forward LSTM and a backward LSTM. Thus, an input sequence is processed in both directions (forward and backwards), and the outputs of the forward and backward LSTM cells are concatenated to the input sequence before being passed to the decoder. The following section explains the architecture of the decoder and how it can generate the target sentence based on the context vector.

#### Decoder architecture

The decoder aims to generate the target response based on a context vector derived from the encoder. The decoder operates differently during the training and inference phases. During training, the output of the decoder is used to calculate the loss, but this is not fed back into the decoder to generate the next token. Instead, the next token from the actual (ground truth) output is fed into the decoder at each time step. This process is called teacher forcing. Teacher forcing is the practice of using the actual next word in the sequence as the input to the decoder for the next step rather than using the word that the model itself predicted. This can help the model learn more efficiently. In teacher forcing, the actual output from the training dataset at the current time step $y_{t}$ is provided as the input for the next time step $x_{(t+1)}$, instead of the output generated by the network. The output tokens of the decoder are returned during inference only when responses are generated. Thus, during training, the output sequence for a given input sequence is generated by providing words of actual output through the teacher forcing technique. However, at the test time (inference time), the output sequence for a given input sequence is unknown. Therefore, the output token generated at time step *t* is the input for $t+1.$

In the decoding phase, the token $<SOS>$ is given as input to the RNNs along with the context vector in the first timestep. The $<SOS>$ token indicates the start of decoding and generates the first word of the chatbot response by looking at the context vector. This decoding likely generates ”Not” as the first output of the RNNs. At the next timestep, ”Not” is given as input along with the hidden state from the previous timestep. This step generates the output ”so”. This output generation continues until a unique $<EOS>$ token is reached or a maximum number of tokens occurs. Considering the context vector (*c*) and all previously predicted outputs $\left\{ {y_1, y_2, y_3, \ldots , y_{t-1}} \right\}$ the decoder is trained to predict the next token $y_t$. This prediction is the maximum likelihood estimate of $y_t$. The prediction is given by *y*, the output vector, and *c*, the context vector. Thus, *p*(*y*) is computed as in Eq. (3).

22 $$\begin{aligned} p(y) = \prod _{t=1}^{T} p\left( y_t \mid y_1, y_2, y_3, \ldots , y_{t-1}, x_t\right) \end{aligned}$$

The token is generated with a conditional probability for each timestep *t* by Eq. (4).

23 $$\begin{aligned} p(y_{t}|y_{1},y_{2},y_{3},...y_{t-1},x_{t})= g(y_{t-1},s_{t},c_{t}) \end{aligned}$$

where $g(\cdot )$ is a softmax function and $s_{t}$ is the hidden state of the decoder at timestep *t*, which can be computed by Eq. (5).

24 $$\begin{aligned} s_{t}= f_{\text {enc}}(s_{t-1},y_{t-1},c_{t}) \end{aligned}$$

The encoder and decoder are trained together to maximize the conditional log-likelihood for the actual output, where the parameter is included in the output utterance, as shown in Eqs. (6) and (7).

25 $$\begin{aligned} \theta&=argmax\ \sum _{n=1}^{N}\sum _{t=1}^{T_n}{\log {p}\left( y_t^n\big | y_{t-1}^n,x^n\right) } \end{aligned}$$

26 $$\begin{aligned} \theta&=argmax\left( \log {p}\left( Y\big | X\right) \right) \end{aligned}$$

The negative log-likelihood loss can be computed as in Eq. (8).

27 $$\begin{aligned} L_{LL}=\ -\frac{1}{N}\sum _{n=1}^{N}\sum _{t=1}^{T_n}{\log {p}\left( y_t^n\big | y_{t-1}^n,\ x^n\right) } \end{aligned}$$

where $\theta$ denotes the model parameters, $\left( x^n,\ y^n\right)$ is nth training pair of sentences and $T_n$ is the length of the nth target sentence $\left( y_n\right)$. Considering the model parameters, the cross-entropy loss for the negative loss probability can thus be calculated as in Eq. (9)

28 $$\begin{aligned} L_{CE\ }\theta =\ -\sum _{t=1}^{T}\log {p\left( y_t\big | y_{1\ },\ y_{2\ },\ y_{3\ }\ldots \ldots ..y_{t-1\ },\ X\right) } \end{aligned}$$

## Appendix B

The greedy search involves considering the word with the highest probability at each timestep. On the other hand, beam search explores a set of possible output sequences by considering multiple hypotheses simultaneously, employing a beam width parameter to control the number of hypotheses to consider at each timestep. In this work, beam search is further improved by incorporating a penalty mechanism to alleviate issues related to over-generating or penalising longer sequences. This augmented version of beam search aims to balance precision and diversity in the generated output sequences. The effects and implications of these inference techniques, including greedy search, beam and improved beam search with a penalty, are thoroughly examined and analyzed in the context of the proposed neural models. These inference techniques serve as important components in the generation of output sequences, enabling the proposed neural models to produce accurate and coherent predictions. Further discussions on these inference techniques are provided in the subsequent sections.

### Greedy search decoding (GSD)

Greedy search decoding (GSD) is a commonly used technique in inference due to its simplicity, speed, and ability to provide suitable answers, and thus has become the standard technique in practice. It involves selecting the word with the highest probability at each timestep, based on the current input and previous output to construct the sequence as presented in Algorithm 2. The generated token is then fed back into the model to predict the next token, and this process continues until the end of the sequence is reached.

#### Beam search decoding (BSD)

In addition to GSD, beam search decoding (BSD) is another commonly used method. Unlike GSD, BSD expands all possible next timesteps. It preserves the *k* most probable ones, where *k* is a user-defined parameter which regulates the number of beams over the sequence of probabilities, rather than greedily selecting the most probable next step when the sequence is generated, as presented in Algorithm 3. In other words, beam search works by keeping a list of the best candidate answers at each timestep in the search. At each timestep, the algorithm generates a set of candidate outputs based on the current input and the previous output, and then selects and sorts the top k candidates based on their probability according to the probabilistic model. The top *k* candidates are then used as input for the next step of the search and the process is repeated until the end of the sequence generates tokens or a maximum number of tokens m is reached.

BSD results in much better quality generated output than greedy decoding in some cases because the algorithm considers multiple hypotheses at each step to explore a wider range of possible output sequences, rather than just the single most likely output as in GSD. Thus, the algorithm returns not just one output sequence but *k* different output sequences for which there is a good chance that one of these k sequences is the correct one. In practice, we can say that if m tokens are to be generated, BSD needs $k*m$ decoding runs to generate the output sequences. This can lead to decoding speed and memory usage being computationally intensive. Depending on the size of the pre-trained beam *k*, decoding with beam search is often *k* times slower than greedy decoding. This can make it impractical to use for large output sequences and real-time applications.

#### Beam search with length penalty decoding (BSLPD)

The length normalization method is a small modification or refinement of the BSD algorithm that can help achieve better prediction results, as proposed by Wu et al.^26^, for machine translation tasks. This method aims to encourage longer or shorter responses. Each response hypothesis can be normalized based on its word length and can be represented as in Eq. 13:

29 $$\begin{aligned} {score}_{norm}=\ \frac{{score}_{hy}}{\left( \frac{a+l_{hy}}{b}\right) ^\alpha } \end{aligned}$$

where $l_{hy}$ is the length of words for the current hypothesis; $\alpha$ is a hyperparameter (length normalization coefficient) and its value is often in the range of 0 to 1. When $\alpha$=0, the decoding algorithm falls back to using a vanilla probability (without length normalization) in the search. In general, the values of a and b can be chosen in different ways. If $a=0$ and $b=1$ with $\alpha =1$, then only the length is used as in Eq. (14):

30 $$\begin{aligned} {score}_{hyp}^{norm}\frac{{score}_{hy}}{l_{hyp}^\alpha } \end{aligned}$$

Moreover, scores are normalized according to the following formula from Wu et al.^26^, where $a=0$ and $b=1$ with $\alpha$ adjusted as in Eq. (15) and deduced as in Eq. (16):

31 $$\begin{aligned}&{score}_{hyp}^{norm}\frac{{score}_{hy}}{\left( \frac{5+l_{hy}}{6}\right) ^\alpha } \end{aligned}$$

32 $$\begin{aligned}&lp\left( Y\right) =\ \frac{{(5+\left| Y\right| )}^\alpha }{{(5+1)}^\alpha } \end{aligned}$$

where |Y| is the current target length.

In practice, this refinement method often leads to improved beam search algorithms.
